# Colon Layer and Histological Phenotype Influence mRNA and miRNA Expression in Colon Samples of the DSS Mouse Model

**DOI:** 10.3390/cells15181677

**Published:** 2026-09-16

**Authors:** Ana Unkovič, Aleš Belič, Emanuela Boštjančič, Tanja Mrak, Martina Perše

**Affiliations:** 1 Medical Experimental Centre, Faculty of Medicine, University of Ljubljana, 1000 Ljubljana, Slovenia; 2 Statistics and Modelling, Technical Development Biologics, Novartis Technical Research & Development, Novartis Pharmaceutical Manufacturing LLC, 1000 Ljubljana, Slovenia; 3 Institute of Pathology, Faculty of Medicine, University of Ljubljana, 1000 Ljubljana, Slovenia; emanuela.bostjancic@mf.uni-lj.si; 4 Slovenian Forestry Institute, Večna pot 2, 1000 Ljubljana, Slovenia

**Keywords:** DSS-induced colitis, formalin-fixed paraffin-embedded tissue, laser microdissection, spatial heterogeneity, histology-guided analysis, multivariate modelling, gene expression, miRNA

## Abstract

**Highlights:**

**What are the main findings?**

• Histological phenotype, anatomical layer, and colitis phase significantly shape molecular expression patterns and interrelationships among inflammatory and regulatory RNAs.
• Whole colon samples do not consistently reflect RNA expression in colon mucosa or histologically defined mucosal microenvironments.

**What are the implications of the main findings?**

• Histology-guided LMD combined with multivariate modelling provides substantially greater spatial and mechanistic resolution than conventional whole-tissue molecular analysis.
• Archived FFPE animal tissues are an underused resource that can support retrospective, spatially resolved molecular studies and increase scientific output without additional animal use.

**Abstract:**

Spatial heterogeneity is increasingly recognized as a major determinant of molecular responses in inflammatory bowel disease, yet molecular analyses in dextran sulfate sodium (DSS)-induced colitis commonly rely on whole-colon measurements that may obscure localized biological processes. We investigated how colon layer, histological phenotype, and colitis phase influence RNA expression by combining archived formalin-fixed paraffin-embedded (FFPE) colon tissue, histology-guided laser microdissection (LMD), targeted RT-qPCR, and multivariate modelling. Expression of five mRNAs (*Tnf*, *Tnfr1*, *Tnfr2*, *Duox2*, and *iNos*) and three miRNAs (*miR-223-3p*, *miR-181a-5p*, and *miR-3473b*) was analyzed in whole-colon sections, individual colon-wall layers, and histologically defined mucosal microenvironments. RNA expression varied according to anatomical layer, histological phenotype, and colitis phase, with the most pronounced molecular changes observed in erosive mucosa. Although several RNAs showed similar overall trends, whole-section measurements did not consistently reflect the magnitude or spatial distribution of RNA expression observed in individual layers and histological microenvironments. Multivariate modelling further revealed context-dependent interrelationships among the analyzed mRNAs and miRNAs and enabled these molecular patterns to be evaluated in relation to histological, anatomical, and temporal variables. These findings demonstrate that spatial and histological context can substantially influence the interpretation of targeted molecular measurements in DSS colitis and that whole-colon analysis may miss spatially restricted molecular changes. Histology-guided analysis of archived FFPE tissue, combined with multivariate modelling, therefore provides a complementary approach for resolving tissue heterogeneity and extracting additional biological information from existing animal tissue repositories, potentially reducing the need for additional animal experiments when appropriate archived material is available.

## 1. Introduction

Inflammatory bowel disease (IBD), encompassing Crohn’s disease (CD) and ulcerative colitis (UC), comprises heterogeneous, chronic, relapsing inflammatory disorders arising from complex interactions among genetic susceptibility, dysregulated immune responses, intestinal microbiota, epithelial barrier dysfunction, and environmental factors [1,2]. This heterogeneity extends from clinical phenotypes to cellular and molecular processes that differ among intestinal regions, tissue compartments, cell populations, and disease stages. Despite advances in therapy, treatment responses remain variable, reflecting persistent gaps in our understanding of IBD pathophysiology [3,4].

Recent single-cell and spatial profiling technologies have revealed that intestinal inflammation is organized within distinct epithelial, immune, and stromal niches [5,6,7]. Whereas conventional bulk analyses average molecular signals across heterogeneous tissue and dissociation-based single-cell approaches lose their original spatial context, spatial methods preserve tissue architecture and relate molecular changes to defined histological microenvironments [5]. In human IBD, such approaches have identified spatially organized cellular networks in CD [6] and distinct molecular profiles associated with histologically defined regions in FFPE colonic tissue [7]. These findings emphasize that understanding intestinal inflammation requires consideration not only of the identification of altered molecular pathways but also of the spatial and temporal context in which these alterations occur.

This principle is particularly relevant to experimental colitis. DSS-induced colitis is a well-established and widely used model to investigate IBD pathogenesis and evaluate therapeutic strategies [8,9,10]. However, DSS-induced colitis exhibits spatial and histological heterogeneity, making it particularly suitable for investigating whether molecular measurements obtained from whole-colon tissue accurately represent responses occurring within specific anatomical layers and histological phenotypes. Disease severity, distribution, histopathology, and course vary with genetic background, microbiological status, experimental conditions, DSS protocol, and disease phase [8,9,10,11,12,13,14,15,16]. Inflammation may affect the entire colon or occur in a patchy distribution, with lesions ranging from mild mucosal inflammation to severe epithelial erosion and involvement of deeper layers of the intestinal wall [9,11,12]. Importantly, these differences can occur not only between animals but also between regions of the same colon [13,14,15], making spatial heterogeneity an intrinsic feature of the model [9,12,13,14]. Recent spatial and longitudinal analyses of other experimental colitis models further demonstrate that epithelial, immune, and stromal responses form distinct spatial and temporal inflammatory programmes [17].

Despite this heterogeneity, molecular analyses in DSS studies commonly rely on predefined colon segments or homogenized tissue, while histopathological assessment is performed on separate samples [18,19]. Consequently, molecular measurements represent averaged signals from multiple cell populations and anatomical layers and may not correspond to the histological phenotype used to characterize disease severity. This limitation is particularly relevant when lesions are focal and not macroscopically identifiable [9,12,20]. In human IBD, by contrast, endoscopically guided sampling and histopathological evaluation are closely integrated in disease assessment [21,22,23]. This is particularly relevant in UC, where histological assessment captures both inflammatory activity and structural mucosal changes. Characteristic findings include inflammatory cell infiltration, crypt architectural distortion, cryptitis and crypt abscesses, mucin depletion, and, in active disease, epithelial injury, erosion, and ulceration. Although inflammation in UC is predominantly mucosal and classically continuous, its severity and microscopic appearance may vary with disease activity and across affected regions [21,22,23]. The DSS model reproduces several features of active mucosal injury and inflammation observed in UC, although it does not recapitulate the full chronic histopathological spectrum of the human disease [9]. Since the histological characteristics of the colon segment used for molecular analysis are usually unknown in DSS colitis studies, sampling strategies that do not account for local histology may introduce variability and obscure biologically relevant spatial differences.

Archived formalin-fixed paraffin-embedded (FFPE) tissue provides an opportunity to bridge this methodological gap. FFPE archives are extensively exploited in human pathology and increasingly support spatial molecular profiling while preserving tissue architecture [5,7]. In contrast, FFPE repositories generated during animal experiments remain comparatively underused for molecular research. We previously demonstrated that archived DSS FFPE colon samples provide reliable mRNA and microRNA (miRNA) measurements and allow molecular findings to be related directly to the histological phenotype of the same tissue [20]. Their reuse can therefore improve the histological contextualization of molecular data while increasing the scientific information obtained from existing animal experiments without additional animal use.

Laser microdissection (LMD) further enables anatomically and histologically defined regions to be isolated directly from FFPE sections under microscopic visualization [24]. This is particularly relevant to the intestinal wall, where distinct tissue layers and inflammatory microenvironments contain different cellular populations and may exhibit different molecular responses [25,26,27,28].

In the present study, we combined LMD of archived FFPE whole-colon sections with targeted RT-qPCR and multivariate modelling to investigate the spatial and temporal organization of molecular responses in DSS colitis. RNA expression was compared between whole-colon sections, individual colon layers, and histologically defined mucosal microenvironments across different phases of colitis. This design enabled us to evaluate the contributions of histological phenotype, anatomical layer, and colitis phase to molecular expression patterns and, simultaneously, to examine interrelationships among the analyzed inflammatory, oxidative-stress-related, and miRNAs. To our knowledge, this is the first animal study combining histology-guided spatial sampling of FFPE tissue with multivariate modelling to simultaneously address these sources of biological variation and molecular interrelationships. Our findings demonstrate marked spatial heterogeneity and show that whole-section measurements do not consistently reflect layer- or lesion-specific expression, while illustrating how archived FFPE repositories can yield additional biological information through integrated spatial and multivariate analysis. The term anatomical layer refers to the major layers of the colon wall (mucosa, submucosa, muscularis propria, and serosa), whereas histological phenotype refers to the microscopically defined tissue appearance (normal, inflammation, erosion, or squamous overgrowth). The term histological microenvironment denotes a spatially defined tissue region characterized by a specific histological phenotype and selected for molecular analysis by laser microdissection. These terms are used consistently throughout the manuscript to distinguish anatomical organization from local histological characteristics.

## 2. Materials and Methods

### 2.1. Selection Process of FFPE Colon Samples

**Figure 1:**
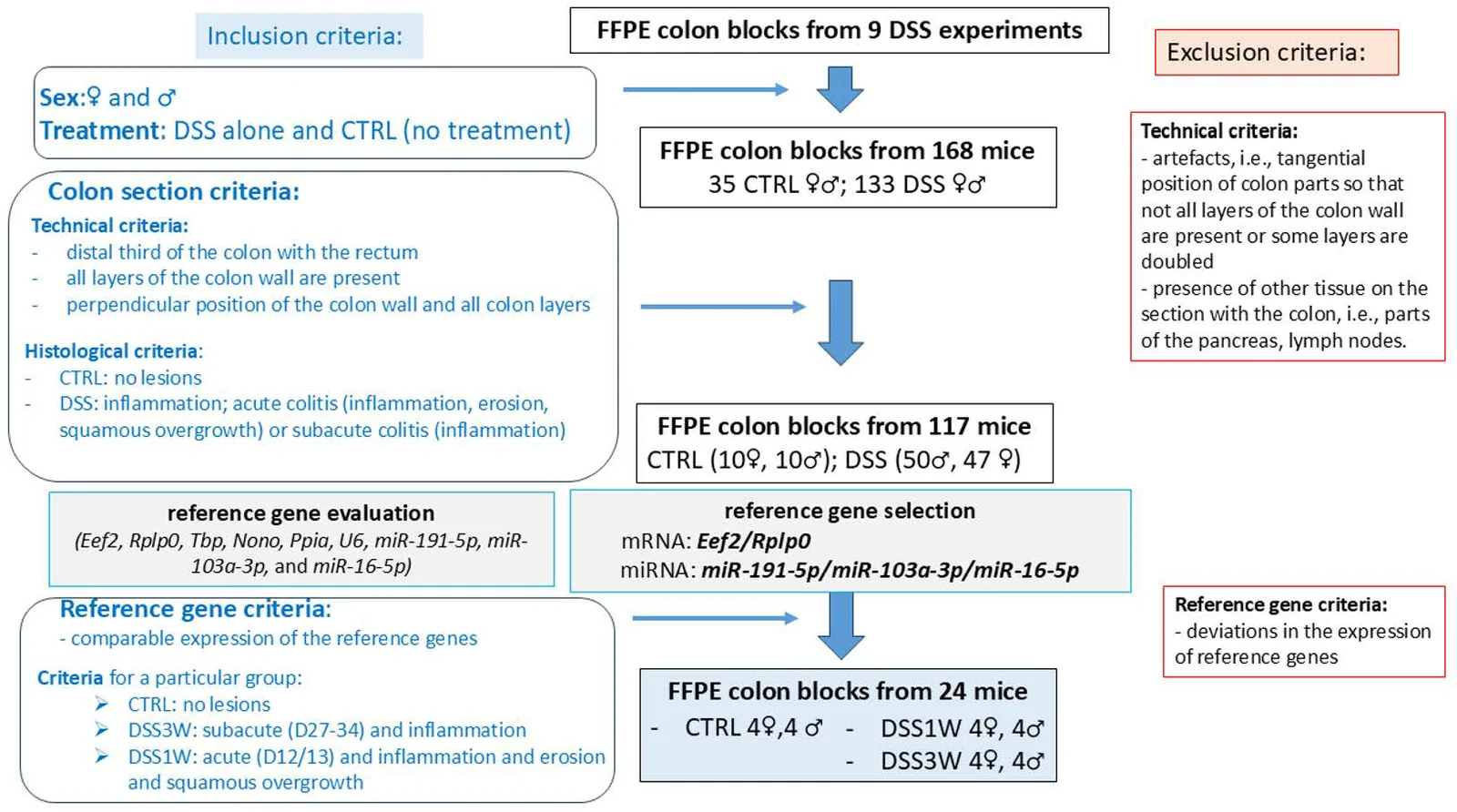
Selection process of FFPE colon samples. Legend: ♂—males; ♀—females; CTRL—control group; DSS—DSS group; DSS1W—DSS acute group; DSS3W—DSS subacute group; FFPE—formalin-fixed paraffin-embedded.

FFPE colon blocks were selected from the archive of the Medical Experimental Centre and originated from nine DSS experiments performed between 2010 and 2014. A multistage selection process based on experimental, technical, histological, and reference gene expression criteria was used to identify relevant FFPE colon sections for our study (Figure 1).

In the first step, only FFPE colon blocks from male and female C57BL/6JOlaHsd mice that had either received DSS alone or remained untreated as controls (CTRL) were in line with the study aims, resulting in an initial set of 168 mice. FFPE colon blocks were cut into 2–5 µm sections and stained with the Kreyberg–Jareg method for subsequent technical and histological evaluation.

In the second step, FFPE colon sections from the distal third of the colon, including the rectum, were assessed according to predefined technical and histological criteria. FFPE colon sections were included when all layers of the colon wall were present and appropriately oriented. FFPE colon sections showing technical artefacts such as tangential orientation resulting in missing or duplicated colon-wall layers or containing tissue from other abdominal organs (e.g., parts of the pancreas, lymph nodes) were excluded. Histologically, FFPE colon sections from the control group (CTRL) were required to show histologically normal colon with no signs of injury or inflammation, whereas FFPE colon sections from DSS-treated mice were required to show colitis. Depending on the disease phase, FFPE colon sections from DSS-treated groups showed colitis with varying intensities, extents, and degrees of inflammation, as well as other colon lesions, such as erosion and squamous overgrowth. DSS1W represents the acute/early post-DSS phase, whereas DSS3W represents the later subacute post-DSS phase; their distinct histological characteristics are described in the Results and illustrated in Figures S2 and S3. Following technical and histological assessment, FFPE colon blocks from 117 mice fulfilled these criteria.

In the third step, candidate reference genes (*Eef2*, *Rplp0*, *Tbp*, *Nono*, *Ppia*, *U6*, *miR-191-5p*, *miR-103a-3p*, and *miR-16-5p*) were evaluated in the remaining FFPE colon sections, as previously described [20]. Based on this evaluation, *Eef2* and *Rplp0* were selected as reference genes for mRNA, and *miR-191-5p*, *miR-103a-3p*, and *miR-16-5p* for miRNA. FFPE colon sections showing comparable expression of the selected reference genes were retained. Finally, FFPE colon blocks from 24 mice (12 females and 12 males) met all selection criteria and were included in the study. The selection process, including inclusion and exclusion criteria, is shown in Figure 1.

#### Animals, DSS Experimental Protocol and FFPE Archive

In DSS experiments, 7–9-weeks-old female or male C57BL/6JOlaHsd mice (Harlan, Italy or Medical Experimental Centre, Ljubljana, Slovenia) were used. Mice were housed under the following conditions: 5–8 mice per cage (Ehret, Mahlberg, Germany; 825 cm^2^ floor area), bedding (Lignocel ¾, J. Rettenmaier & Soehne GmbH, Rosenberg, Germany), cellulose towels, and under controlled environmental conditions: 12 h light/dark cycle (7 a.m./7 p.m.). Mice had constant access to tap water and rodent diets (Altromin 1324, Altromin Spezialfutter GmbH, Lage, Germany or Teklad 2018, Harlan Laboratories S.r.l., San Pietro al Natisone, UD, Italy). To induce colitis, DSS (molecular weight 40 kDa, TdB Consultancy AB, Uppsala, Sweden) was added to drinking water for 4–6 days. The DSS solution, with a concentration of 2–3%, was freshly made every morning. Controls received drinking water alone. After DSS treatment (4–6 days), mice received drinking water for different time periods (1 week, 3–4 weeks). Mice were euthanized with CO_2_. Autopsy, sampling, and formalin fixation were performed under the same environmental conditions using the same necropsy protocol. All internal organs (except the central nervous system) were examined. The whole colon was removed, wide open, formalin-fixed, and paraffin-embedded. The Medical Experimental Centre stored FFPE blocks at room temperature in their archives. All paraffin blocks were stored under the same conditions. All animal experiments were performed in accordance with Directive 2010/63/EU and Slovenian legislation (permit No. 34401-17/2010/15, issued on 15 December 2010 and 34401-54/2012/5, issued on 14 January 2013 from the Food Safety, Veterinary and Plant Protection Administration).

### 2.2. Laser Microdissection (LMD) Method and Staining

LMD PALM MicroBeam (inverted Zeiss microscope and PALM laser light source (λ = 355 nm), at the Slovenian Forestry Institute) was used on 7 μm whole FFPE colon sections to isolate compartments (colon layers) of interest under microscopic visualization (400× magnification). For this purpose, FFPE colon sections were placed on nuclease and human nucleic acid free polyethylene naphthalate (PEN) membrane slides (Leica, MicroDissect, Herborn, Germany) and stained with Haris’s haematoxylin according to the following protocol: 5 min xylene, 3 min 100% ethanol, 3 min 95% ethanol, 1 min 70% ethanol, rinse with nuclease-free water, 5 min of double Haris’s haematoxylin, rinse with nuclease-free water, 5 min alcian blue, rinse with nuclease-free water, 2 s picrofuxin, rinse with 100% ethanol, stored in xylene (up to 2 h) and air-dried before LMD. Colon layers (such as mucosa, submucosa, muscularis propria, and serosa) of histologically selected colon section regions (normal, inflammation, erosion, squamous) were microdissected and varied in total area from 2147 to 2,549,717 μm^2^. Each FFPE colon section layer isolated by LMD was collected in a 0.5 μL adhesive cap (Carl Zeiss Microscopy GmbH, Göttingen, Germany) and stored at −20 °C until further processing.

### 2.3. RNA Isolation from Whole FFPE Colon Sections and LMD Samples

The commercially available miRNeasy FFPE Kit (Qiagen, Hilden, Germany) was used to isolate total RNA according to the manufacturer’s instructions. Isolation of total RNA was performed from whole FFPE colon sections (four 10 µm thick sections per block were cut in an Eppendorf tube) and from colon layers isolated by LMD.

All reagents, except ethanol and hexadecane (Sigma-Aldrich; Merck, Kenilworth, NJ, USA), were supplied by Qiagen. The quantity and quality of isolated RNA were determined using NanoDrop One (Thermo Fisher Scientific, Waltham, MA, USA). Absorbance was measured spectrophotometrically at 260 nm to determine RNA quantity, and impurities were detected at 230 and 280 nm. As all our LMD samples had RNA concentrations around 20 ng/µL or lower, the purity ratios were unreliable according to the NanoDrop One. Whole-colon sections had an absorption ratio A260/280 of 1.8 or higher than 1.8, indicating that RNA was of good quality.

### 2.4. mRNA and miRNA Analyzed in FFPE Colon Sections and LMD Samples

To investigate the influence of sampling strategy on molecular readouts in DSS colitis, we analyzed a panel of several well-characterized inflammatory, oxidative-stress-related, and regulatory RNAs. The analyzed RNAs were selected based on previous findings in DSS-induced colitis and their complementary roles in inflammatory and regulatory responses. These included oxidative stress-related molecules inducible nitric oxide synthase (*iNos*) and dual oxidase 2 (*Duox2*), which participate in intestinal redox regulation and inflammatory responses [29,30,31], tumour necrosis factor (*Tnf*) and its receptors *Tnfr1* and *Tnfr2*, which mediate context-dependent inflammatory and immunoregulatory signalling [32,33,34], and miRNAs implicated in inflammatory regulation and TNF-associated pathways, including *miR-223-3p*, *miR-181a-5p*, and *miR-3473b* [35,36,37,38,39]. Together, this targeted panel was designed to capture complementary aspects of the molecular response to intestinal inflammation rather than to provide comprehensive pathway coverage.

#### Selection of Reference Genes

A combination of *Eef2/Rplp0* was used as a reference gene for mRNA, and a combination of *miR-191-5p/miR-103a-3p/miR-16-5p* was used as a reference gene for miRNA. Evaluation and selection of reference genes for mRNA and miRNA were made on FFPE whole-colon sections from 117 mice of both sexes from 9 different DSS experiments to ensure technically comparable normalization across archived FFPE samples and were independent of the expression of the target RNAs investigated here (described elsewhere [20]). Briefly, for mRNA, the expression stability of five candidate reference genes (*Eef2*, *Tbp*, *Rplp0*, *Nono*, *Ppia*) were evaluated, and for miRNA, the expression stability of four reference genes (*miR-191-5p*, *miR-103a-3p*, *miR-16-5p* and *U6*) were evaluated. Based on the obtained quantification cycles (Cq) of the reference genes, the analysis of the stability of their expression, and multivariate analysis.

Official mouse gene symbols follow Mouse Genome Informatics (MGI) nomenclature. For readability, the commonly used abbreviations *Tnfr1*, *Tnfr2*, and *iNos* are used throughout the text for *Tnfrsf1a*, *Tnfrsf1b*, and *Nos2*, respectively.

Mature miRNA names are given in their commonly used abbreviated form with the species prefix mmu- omitted for readability. miRBase accession numbers correspond to the targets of the QIAGEN miRCURY LNA miRNA PCR assays used. For miR-191-5p, miR-103a-3p, and miR-16-5p, the assays are human-designated and therefore list human miRBase accession numbers; however, the corresponding human and mouse mature miRNA sequences are identical, and the assays are specified for multiple species, including *Mus musculus*. See Supplementary Tables S1–S3 for sequence and assay details.

### 2.5. Reverse Transcription and Quantitative Real-Time Polymerase Chain Reaction (RT-qPCR)

RT-qPCR reactions for mRNA were made using Luna^®^ Universal One-Step RT-qPCR Kit (New England Biolabs, Inc., Ipswich, MA, USA) and hydrolysis probes (TaqMan^®^ detection probes, Applied Biosystems, Foster City, CA, USA). All reactions were performed in a QuantStudio ™ 7 Pro (Applied Biosystems, Waltham, MA, USA). In each individual reaction for LMD, there were 2 ng of RNA and 10 ng of RNA for whole tissue sections in a final volume of 10 µL. The RT and PCR conditions were as follows: 55 °C for 10 min, 95 °C for 1 min, 45 cycles at 95 °C for 15 s and at 60 °C for 1 min. For each hydrolysis probe, negative controls (with no RNA template) were used. All RT-qPCR reactions were made in duplicates. The commercial TaqMan assays that generated short amplicons (64–81 bp) were used, thereby limiting the effect of FFPE-associated RNA fragmentation on amplification (Table 1). No additional probe-specific optimization of the RT-qPCR cycling conditions was performed. Cq values were determined using the automatic threshold setting in the QuantStudio software and were not adjusted according to the presumed degree of RNA degradation. Technical duplicates were averaged for subsequent analysis; reactions showing excessive variability between replicates (SD > 1 Cq) were repeated or excluded.

In comparison to mRNA, RT reactions for miRNA were performed separately from qPCR reactions in the PTC-200 Thermal Cycler (MJ Research, Waltham, MA, USA). For RT reactions, miRCURY LNA RT Kit (Qiagen, Hilden, Germany) was used. All reactions were made according to the manufacturer’s instructions. Synthetic RNA spike-in UniSp6 was added to the reverse-transcription reactions according to the manufacturer’s protocol and subsequently quantified to monitor the efficiency and consistency of reverse transcription. In each reaction, 10 ng of RNA was used. The RT conditions were as follows: 60 min at 42 °C, followed by 5 min at 95 °C. cDNAs were stored at −20 °C. qPCR reactions were made using miRCURY LNA SYBR^®^ PCR Kit (Qiagen, Hilden, Germany) and miRCURY LNA miRNA PCR Assays (Qiagen, Hilden, Germany), in a final reaction volume of 10 µL. All reactions were performed in a QuantStudio ™ 7 Pro (Applied Biosystems, Waltham, MA, USA). In each reaction for LMD samples, 3 µL of 1:20 diluted cDNA (or 1:60 diluted cDNA for tissue sections) was used. The qPCR conditions were as follows: 2 min at 95 °C, 40 cycles at 95 °C for 10 s and at 56 °C for 1 min, followed by melting curve analysis at 60–95 °C. For each miRNA PCR Assay, negative controls (with no RNA template) were used. All qPCR reactions were made in duplicates. Cq values were determined using the automatic threshold setting in the QuantStudio software. Technical duplicates were averaged for subsequent analysis, applying the same quality-control criteria as for mRNA analysis.

### 2.6. Amplification Efficiency

Amplification efficiency was determined for each mRNA and miRNA assay using pooled RNA from FFPE colon sections, separately for the control and DSS groups. For this purpose, standard curves consisting of 5-point serial dilutions of pooled RNA samples contained equal amounts of RNA from individual samples within each group. Five-point serial dilution curves were prepared using 5-fold dilutions for mRNA assays and 4-fold serial dilution for miRNA assays. Each dilution was analyzed in triplicate. Standard curves were generated by plotting Cq values against the corresponding RNA dilution, and amplification efficiencies were calculated from the slopes of the resulting curves. The coefficient of determination (R^2^) was used to assess the linearity of each standard curve. The amplification efficiencies and R^2^ values obtained for individual mRNA and miRNA assays are presented in Table 1 and Table 2, respectively.

### 2.7. Statistical Analysis

The investigator performing the molecular analyses of the archived FFPE samples and subsequent data analysis was blinded to the original experimental group allocation; samples were analyzed using coded identifiers. Normalization and expression analysis of RT-qPCR mRNA and miRNA data was made according to Latham [40]. Cq values from technical duplicates were averaged before normalization, and the resulting ∆Cq values were used for all statistical analyses of mRNA and miRNA expression. Statistical analysis of the results was made using IBM SPSS Statistics, version 27 (SPSS Inc., Chicago, IL, USA). The Mann–Whitney U test was used to compare ∆Cq values between independent groups, whereas the Wilcoxon signed-rank test was used for comparisons between dependent sample groups. The differences were marked as statistically significant at *p* < 0.05. Excel 2016 (Microsoft Corporation, Redmond, WA, USA) was used for graphical display of the results. For multiple comparisons of effects of histology, the significance limit for *α* was reduced from 0.05 to 0.018 using Benjamini–Hochberg method to reduce false discovery rate [41]. Also, in estimating the effects of histology, ∆Cq values were used in statistical evaluation.

### 2.8. Multivariate Analysis

For multivariate data analysis, the method of partial least squares (PLS) [42,43] was used. PLS was performed in SIMCA 16 (Sartorius Stedim Data Analytics AB, Umeå, Sweden). PLS is a multivariate modelling method in which principal component analysis (PCA) is used to decorrelate model input and output variables, followed by application of multiple regression on decorrelated variables. The method is superior to multiple regression for datasets where model inputs are correlated. The method can rank inputs according to the size of their effects on the outputs via centred and scaled coefficients, where differences between variabilities of inputs is taken into account to enable fair comparison between inputs of different units. Centred and scaled coefficients thus represent relative contributions of model inputs. The data set was separated into training and test data sets in a ratio 6:1, with every seventh row selected as test. SIMCA estimates model coefficients’ statistics by varying training and validation data sets. Coefficients with confidence intervals larger than their mean value are considered non-significant (their value depends only on how training and test data sets are selected) and corresponding inputs can be removed from the model. The PLS models were validated by comparing R^2^, Q^2^, and performing permutation tests. R^2^ is a coefficient of determination and explains how much of the total data variability can be explained by the model and is calculated on the training data set. Q^2^ is a coefficient of determination calculated on the test data set, estimating predictive power of the model. Q^2^ is generally smaller than R^2^, but the difference should not be large for good models. The permutation test mixes input-output data pairs and calculates R^2^ and Q^2^ for the model on the mixed data set. If R^2^ and Q^2^ for the correct input–output data pairs are higher than for the mixed data set, there is some meaningful relation in the data that were captured by the model.

Two models were constructed for each RNA expression. The first model aimed to predict specific RNA expression in whole colon sections with the same RNA expression in colon layers. Additional inputs to the model were sex and histology, so all available samples were described using the same PLS model rather than grouping them by histology and sex, as is required in univariate statistical approaches. The second model described the expression of a specific RNA in all layers using histology, layer information and the expression of all other measured RNAs. Again, the advantage is that we can use a single model for each RNA expression prediction covering all available samples.

## 3. Results

### 3.1. Histological Characteristics of Whole FFPE Colon Sections and LMD Excised Colon Areas

Histological examination demonstrated distinct tissue characteristics between the two DSS groups (Figures S2 and S3). DSS1W, representing the acute/early post-DSS phase, was characterized by pronounced active inflammation and epithelial injury, including extensive erosion with loss of epithelium and crypt architecture, marked inflammatory cell infiltration, and areas of squamous overgrowth extending from the rectum into the adjacent colon. Inflammatory infiltration involved the mucosa and submucosa and was focally transmural. In contrast, DSS3W represented a later subacute phase with persistent but more heterogeneous inflammatory changes. Inflammation was predominantly mucosal, although it extended into the submucosa or deeper layers in some regions. Mucosal morphology ranged from areas with sparse residual crypts, marked mucin depletion, and dense inflammatory infiltration to areas with comparatively preserved crypt architecture and mucin content accompanied by inflammatory infiltration beneath the crypt bases. Squamous overgrowth extending from the rectum into the adjacent colon was also observed. Representative histological features of CTRL, DSS1W, and DSS3W samples are shown in Figures S1–S3.

**Figure 2:**
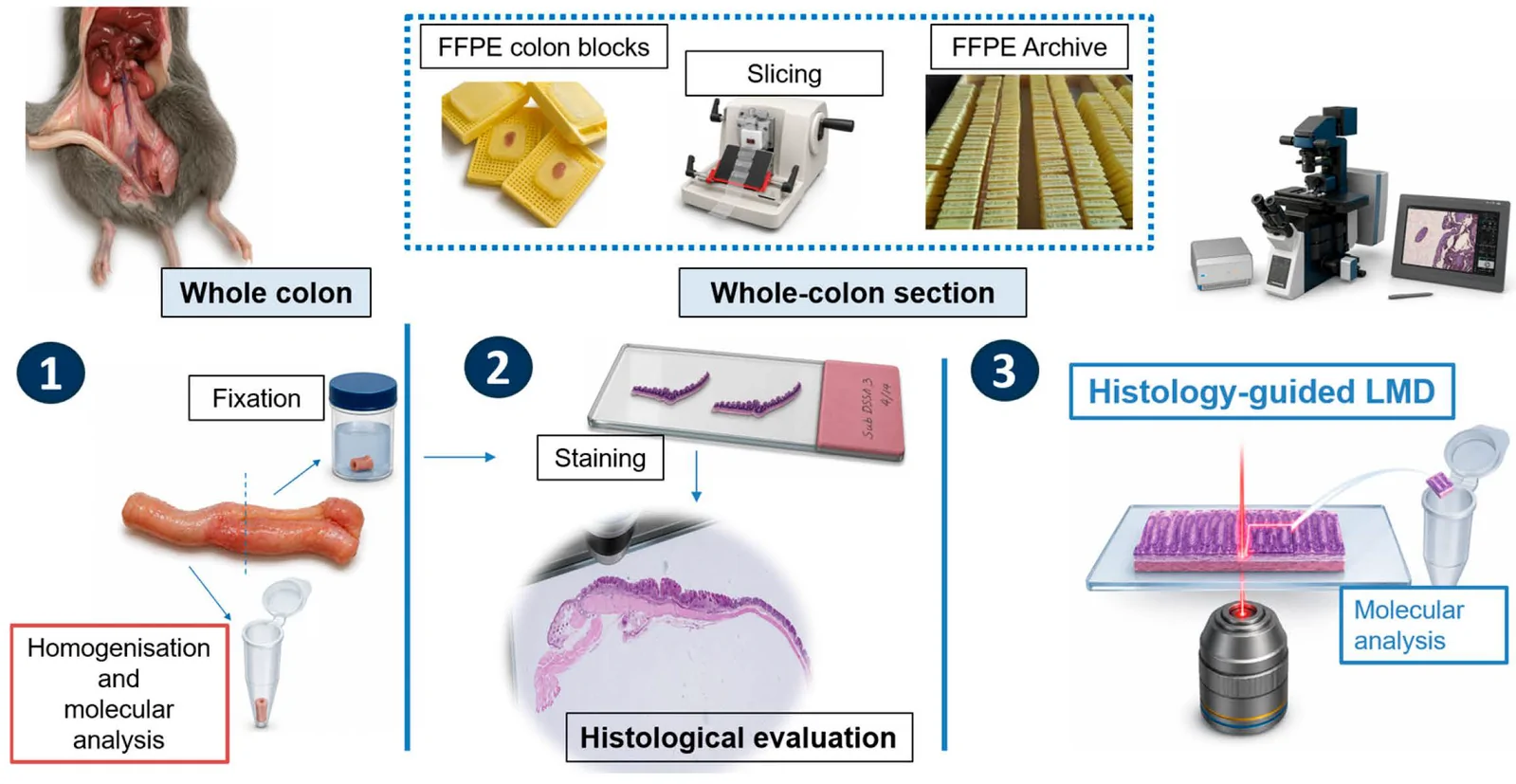
Conventional bulk analyses of homogenized colon tissue versus histology-guided molecular analysis of DSS-induced colitis. (1) In conventional DSS studies, a defined colon segment is commonly collected at autopsy and homogenized for molecular analysis. (2) A separate colon segment is formalin-fixed and paraffin-embedded, sectioned, stained, and evaluated histologically. FFPE blocks generated for histopathological evaluation are commonly retained in archives and represent a potential resource for retrospective molecular studies. (3) In the present study, archived FFPE whole-colon sections were subjected to LMD, enabling the precise isolation of anatomically and histologically defined tissue compartments under direct microscopic visualization. Abbreviations: FFPE, formalin-fixed paraffin-embedded; LMD, laser microdissection.

Using the LMD method, under the microscope (400× magnification), individual colon layers (mucosa, submucosa, muscularis propria, and serosa) of the area of interest (healthy, inflammation, erosion, and squamous overgrowth) were excised from each whole FFPE colon section (Figure 2) of a particular group as follows:

• CTRL whole-colon sections: untreated control mice with a histologically healthy colon (Figure S1). For LMD, colon layers were cut out and taken for further RNA analyses.
• DSS acute whole-colon sections (DSS1W): DSS-treated mice with colitis and the following histological types of lesions: inflammation, erosion, and squamous overgrowth (Figure S2). For LMD, individual layers from each histological phenotype were cut out and taken for further RNA analyses.
• DSS subacute whole-colon sections (DSS3W): DSS-treated mice with histologically confirmed colitis with various intensities of inflammation (Figure S3). For LMD, only areas with mild to moderate inflammation, limited to the mucosa and submucosa, were excised and processed for further RNA analyses.

MiRNAs and mRNAs were detected in all whole FFPE colon sections (24/24 animals) and in almost all LMD mucosa layers, while in other colon layers (submucosa, muscularis propria, and serosa) the Cq-values of mRNAs were often below the detection level or 3–5 cycles higher than the Cq-values of miRNAs [40]. For more details, see Figure S4 or the raw data file.

### 3.2. Whole FFPE Colon Sections Do Not Fully Reflect Mucosal RNA Expression Patterns

**Figure 3:**
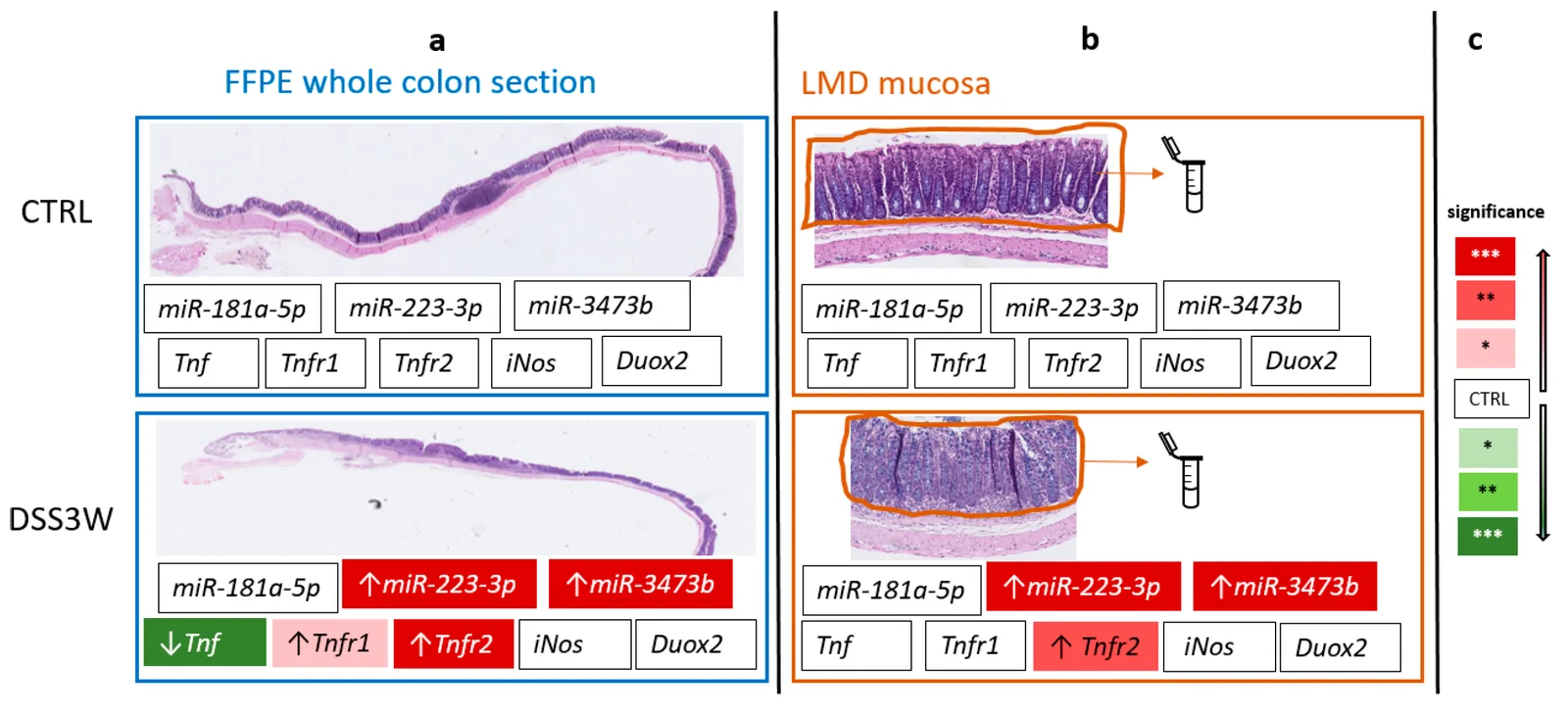
Representative histological pictures and RNA expression patterns in whole FFPE colon sections ((**a**); magnification 4×) and in the mucosa ((**b**); magnification 40×) of the CTRL and DSS3W groups (n = 8 C57BL/6JOlaHsd mice; 4 males and 4 females). (**b**) Mucosa excised by LMD (brown line) and taken for RNA analyses. Kreyberg–Jareg staining. Legend: the green or red colour RNA box indicates a significant difference from the corresponding CTRL group (white colour box), as shown in the colour scale (**c**): * *p* < 0.05, ** *p* < 0.01, *** *p* < 0.001. Statistical method: Mann–Whitney U.

**Figure 4:**
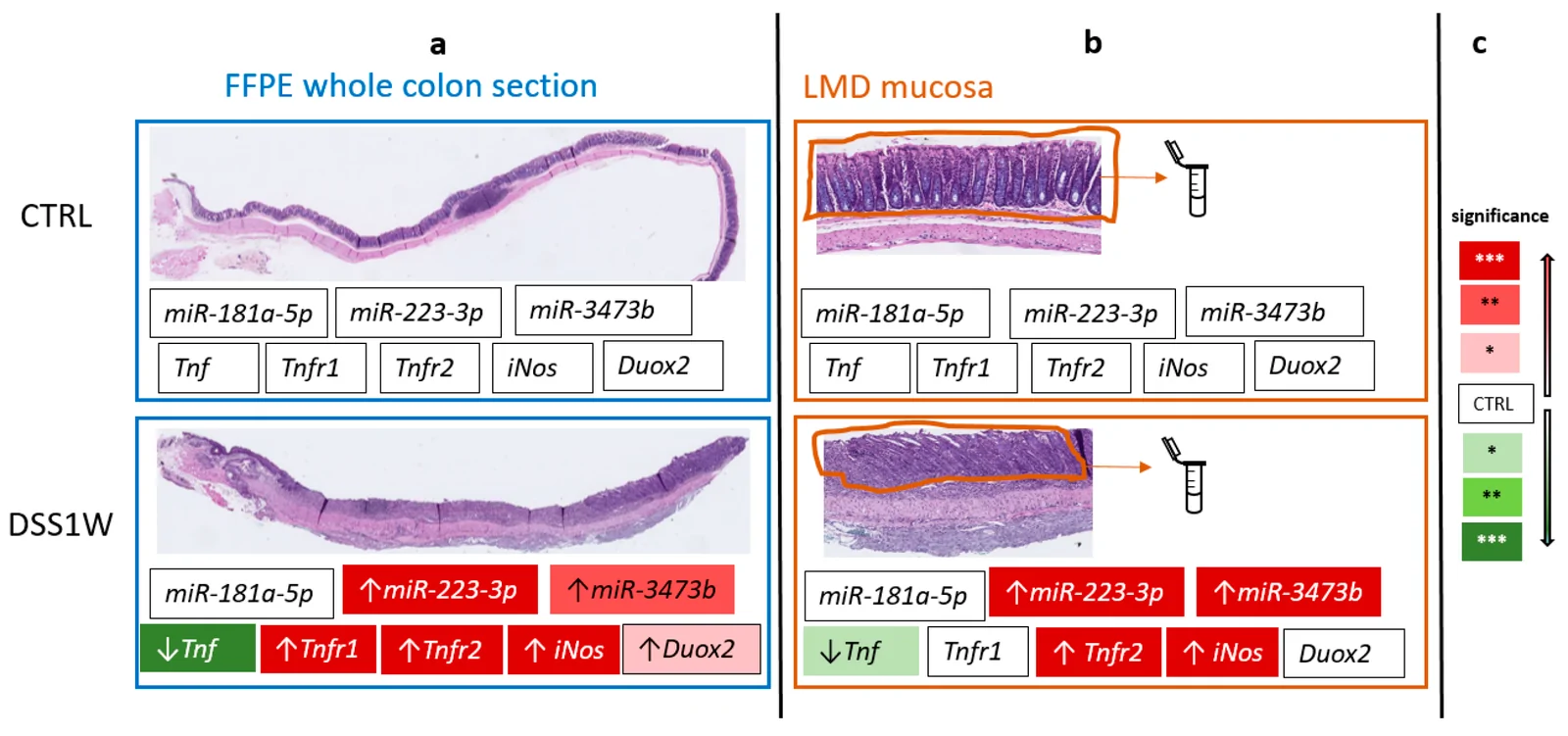
Representative histological pictures and RNA expression patterns in FFPE whole-colon sections ((**a**); magnification 4×) and in the mucosa ((**b**); magnification 40×) of the CTRL and DSS1W groups (n = 8 C57BL/6JOlaHsd mice; 4 males and 4 females). (**b**) Mucosa excised by LMD (brown line) and taken for RNA analyses. Kreyberg–Jareg staining. Legend: the green or red colour RNA box indicates a significant difference from the corresponding CTRL group (white colour box), as shown in the colour scale (**c**): * *p* < 0.05, ** *p* < 0.01, *** *p* < 0.001. Statistical method: Mann–Whitney U.

To investigate whether RNA expression patterns in whole FFPE colon sections reflect those in the mucosa, we compared RNA expression levels between whole FFPE colon sections and mucosa from DSS groups at different phases of colitis (i.e., DSS3W—Figure 3 and Figure S5, and DSS1W—Figure 4 and Figure S6), and the control group.

#### 3.2.1. RNA Expression Patterns in the Subacute Phase of Colitis; DSS3W

In whole FFPE colon sections of the DSS3W group, expression levels of *Tnfr1*, *Tnfr2*, *miR-223-3p* and *miR-3473b* were significantly higher, and expression levels of *Tnf* were significantly lower compared to the control group. For *Duox2*, *iNos* and *miR-181a-5p*, no significant difference was found in whole FFPE colon sections between the control and DSS3W groups (Figure 3a).

Similarly, for *Duox2*, *iNos* and *miR-181a-5p*, no significant difference was found in the mucosa between the control and DSS3W groups, while *miR-223-3p* and *miR-3473b* expression were significantly increased in both mucosa and whole FFPE colon sections of the DSS3W group. Thus, in the mucosa of the DSS3W group, 5 of 8 tested RNAs showed expression patterns that reflected those found in whole FFPE colon sections. In contrast to whole sections, *Tnfr1 and Tnf* in the mucosa showed no significant difference between the DSS3W and the control group. However, in the DSS3W group, the level of significance for *Tnfr2* was lower in the mucosa (*p* < 0.01) than in the whole colon section (*p* < 0.001) (Figure 3b).

#### 3.2.2. RNA Expression Patterns in Acute Phase of Colitis DSS1W

In the whole FFPE colon sections of the DSS1W group, 7 of 8 tested RNAs had significantly different expression; six of them (i.e., *Tnfr1*, *Tnfr2*, *iNos*, *Duox2*, *miR-223-3p* and *miR-3473b*) had significantly higher expression, and surprisingly, *Tnf* had significantly lower expression compared to the control group. For *miR-181a-5p*, no significant difference was found between the control and DSS1W groups (Figure 4a).

Interestingly, in the mucosa of the DSS1W group, only 4 of 8 tested RNAs (i.e., *miR-181a-5p*, *miR-223-3p*, *Tnfr2*, *iNos*) showed expression patterns that reflected those found in the whole-colon sections. Two of 8 tested RNAs (i.e., *Tnfr1* and *Duox2*) showed no significant difference between the control and DSS1W group in the mucosa, while 2 of 8 tested RNAs showed different level of significance in the mucosa compared to the whole-colon section (i.e., *Tnf* mucosa *p* < 0.05 vs. colon *p* < 0.001, and *miR-3473b* mucosa *p* < 0.001 vs. colon *p* < 0.01), showing that in the mucosa expression levels of RNAs and relations between RNAs differ from those observed in the whole colon (Figure 4b).

### 3.3. Colitis Phase Influences RNA Expression Differently in the Mucosa and Whole-Colon Sections

**Figure 5:**
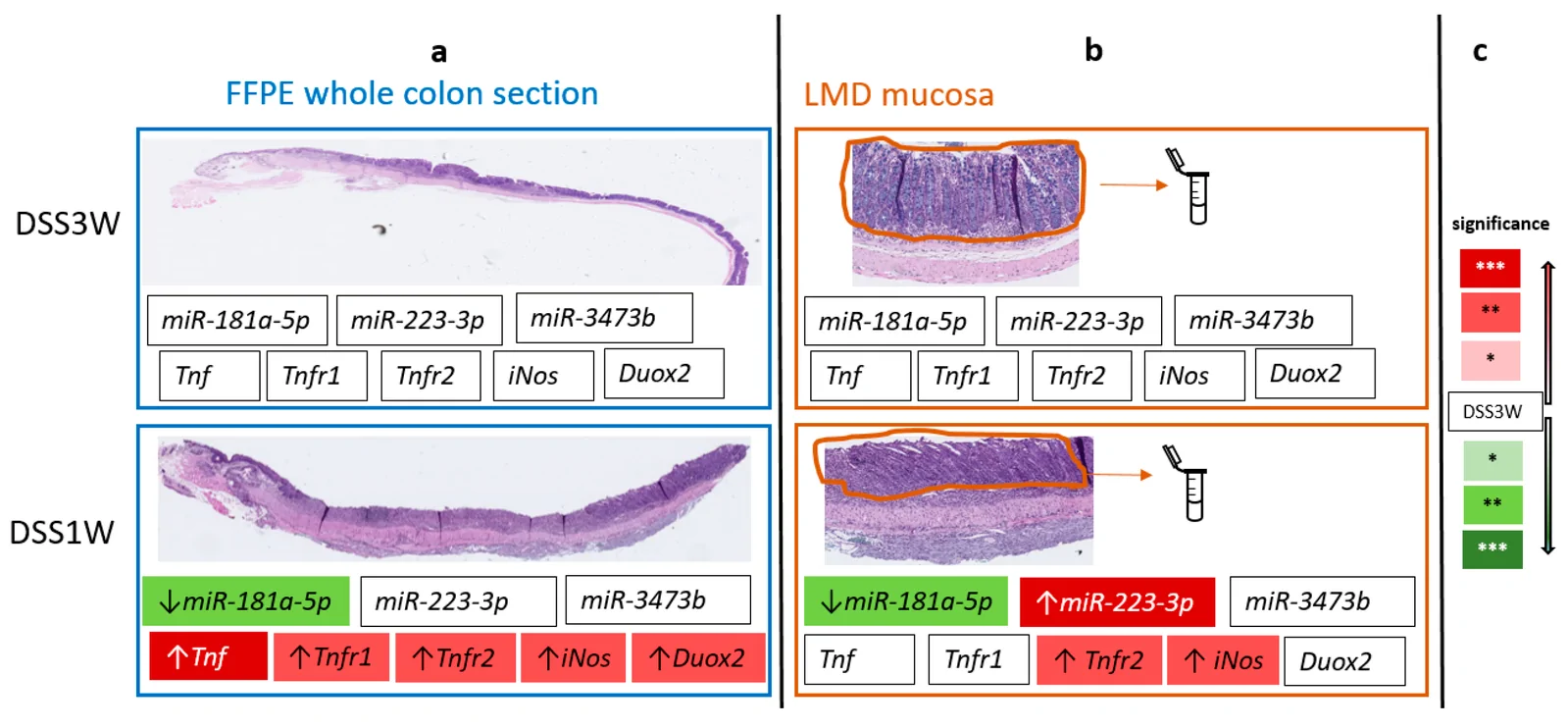
Representative histological pictures and RNA expression patterns in FFPE whole-colon sections ((**a**); magnification 4×) and in the mucosa ((**b**); magnification 40×) of the DSS1W and DSS3W groups (n = 8 C57BL/6JOlaHsd mice; 4 males and 4 females). (**b**) Mucosa excised by LMD (brown line) and taken for RNA analyses. Kreyberg–Jareg staining. Legend: the green or red colour RNA box indicates a significant difference from the corresponding DSS3W group (white colour box), as shown in the colour scale (**c**): * *p* < 0.05, ** *p* < 0.01, *** *p* < 0.001. Statistical method: Mann–Whitney U.

Next, we assessed RNA expression in colon mucosa with a similar histological type (mild to moderate inflammation) but different phases of colitis (i.e., acute DSS1W vs. subacute DSS3W) (Figure 5 and Figure S7).

In the inflamed colon mucosa, *Tnfr2*, *iNos* and *miR-223-3p* had significantly higher expression, and *miR-181a-5p* had significantly lower expression in the DSS1W compared to the DSS3W group, while *Tnf*, *Tnfr1*, *Duox2* and *miR-3473b* showed the same levels of expression in both DSS groups (Figure 5b).

In the whole-colon sections, 6 of 8 tested RNAs had significantly different expression between the two groups (i.e., all mRNAs had significantly higher expression, and *miR-181a-5p* had lower expression in the DSS1W group compared to the DSS3W group) (Figure 5a). However, when comparing RNA expression patterns between the mucosa and the whole-colon section, only 3 of 8 tested RNAs showed a similar pattern in the mucosa as expressed in the whole-colon section of the DSS1W group (i.e., *miR-181a-5p*, *Tnfr2* and *iNos*) (Figure 5).

Results show altered RNA expression between acute and subacute inflammation, confirming that the phase of colitis affects RNA expression, but RNA expression in the whole-colon sections does not reflect expression in the colon mucosa.

### 3.4. Histological Phenotype of Colitis Shapes RNA Expression in the Colonic Mucosa

**Figure 6:**
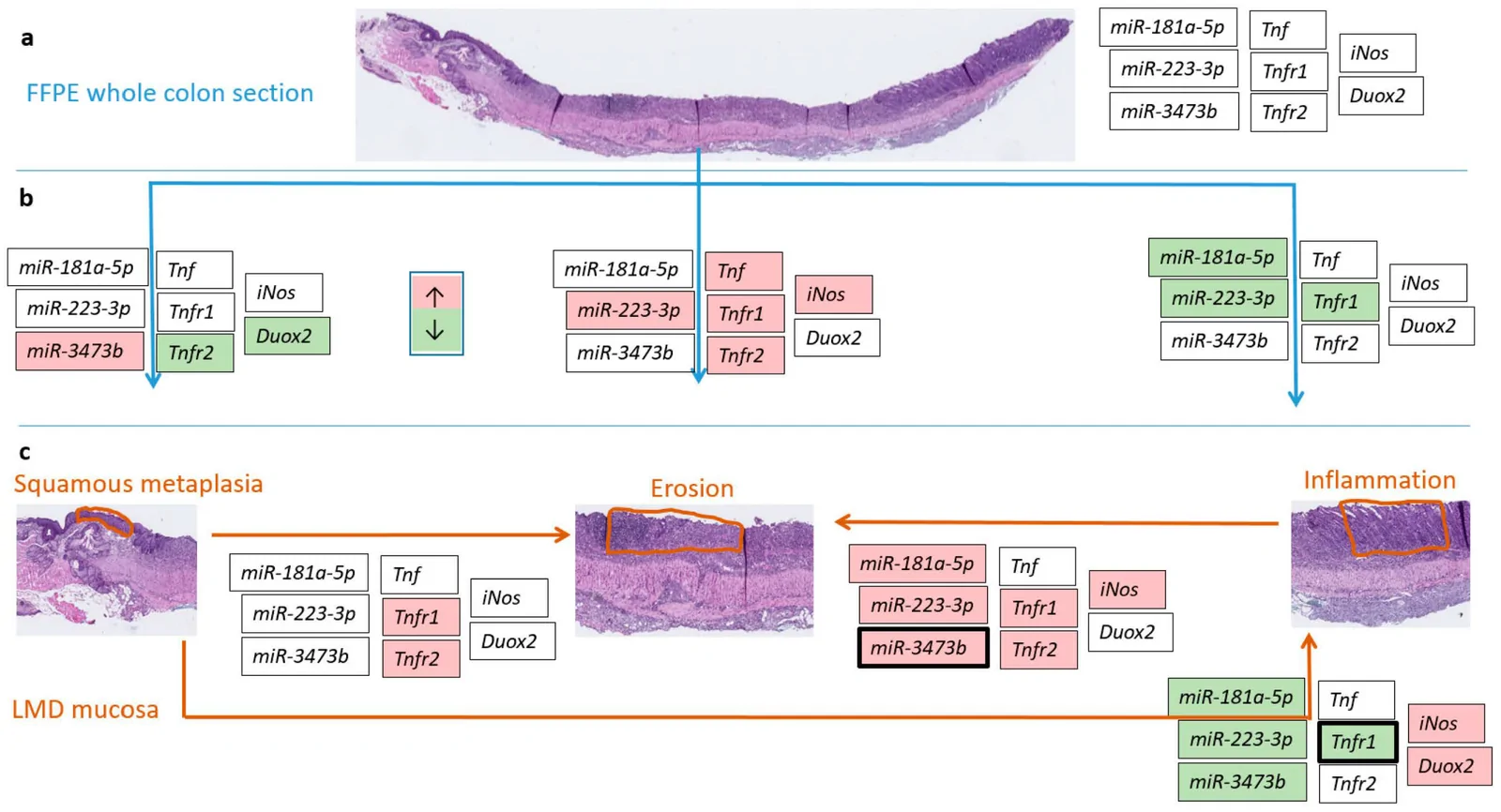
Representative histological pictures and RNA expression patterns in FFPE whole-colon section ((**a**); magnification 4×) and each LMD group (**c**); squamous overgrowth, erosion and inflammation; magnification 40×) of the DSS1W group (n = 8 C57BL/6JOlaHsd mice; 4 males and 4 females). (**b**) Significant RNA changes in the mucosa of each histological phenotype (squamous, erosion, inflammation) when compared to the FFPE whole-colon section. (**c**) Significant RNA changes in the mucosa across histological phenotypes. (**c**) Mucosa excised by LMD (brown line) and taken for RNA analyses. Kreyberg–Jareg staining. Legend: the white colour RNA box denotes no change from the FFPE whole-colon section RNA levels. The red (increased) and green (decreased) colours denote significant differences in RNA levels (*p* < 0.0181) between the two groups linked by an arrow. Significance in RNA levels refers to the group indicated by the arrowhead. Statistical method: Mann–Whitney U.

To get insight into the RNA expression in different histological microenvironments of the colonic mucosa (squamous overgrowth [44], erosion and inflammation), we analyzed the expression of RNA in the mucosa of the DSS1W group. Namely, in the DSS1W group, all analyzed histological types of colitis were present within the same whole-colon section (from the same animal) (Figure 6 and Figure S8). In this way, we excluded the colitis phase and other confounding inter- and intra-experimental factors affecting the DSS model, colitis and RNA expression results [9].

Significant differences in RNA expression were observed among histological phenotypes within the same whole FFPE colon section. The greatest changes were observed in erosion, where 6 of 8 RNA expression levels (i.e., *Tnfr1*, *Tnfr2*, *iNos*, *miR-181a-5p*, *miR-223-3p*, and *miR-3473b*) were significantly higher than those observed in the inflamed mucosa, and 4 of 8 RNA expression levels (i.e., *Tnf*, *Tnfr1*, *Tnfr2*, *iNos*, and *miR-223-3p*) were higher than in the whole-colon sections, and 2 of 8 RNA expression levels (i.e., *Tnfr1* and *Tnfr2*) were significantly higher than in the squamous overgrowth colon mucosa.

In contrast, in the inflamed colon mucosa, 3 of 8 RNAs (i.e., *Tnfr1*, *miR-181a-5p*, and *miR-223-3p*) expression levels were significantly lower than in the whole-colon section, while no significant difference was found for *Tnf*, *Duox2*, *iNos*, and *miR-3473b*. In the mucosa covered by squamous epithelia (overgrowth), *Tnfr2 and Duox2* expression levels were significantly lower, but *miR-3473b* was significantly higher than in whole-colon sections, while for *miR-181a-5p* and *Tnf*, no significant difference was observed. The results show that RNA expression of inflammatory markers in the mucosa is significantly influenced by the histological phenotype of colitis, with the highest expression in erosion.

### 3.5. Compartment-Specific RNA Expression Across the Colon Wall in DSS Colitis

To get insight into the expression patterns of RNA across colon layers (each layer has its own structure, cell types, and function, and thus may contribute differently to the total RNA expression in the colon wall), we analyzed RNA expression in each layer. Since all tested miRNAs and RNAs were detected in all whole-colon sections but not in every colon layer (Figure S4), the RNA expression levels from LMD samples of CTRL, DSS1W and DSS3W were analyzed using multivariate analyses [42,43].

In some cases, we added additional experimental metadata as model inputs (such as DSS treatment, DSS groups, and sex) to account for possible differences coming from experimental conditions. As the samples come from past DSS experiments performed during 2010–2014, combining them might introduce some residual unwanted correlation of influential variables, otherwise compensated by the study experimental design, still present in the results. This way, we were able to at least partially compensate for it with the model. In most cases, the additional experimental information did not show a significant contribution to the mathematical model prediction. However, it was detected as significant in cases where the mathematical model could not identify any significant correlation with key expected contributing factors to the model. This was then interpreted as spurious correlation when the model had a high number of inputs and a low number of samples on which the correlation was estimated. Also, statistical testing of such models provided negative results.

The first set of multivariate models tested, separately for each of the eight analyzed RNA, whether whole-colon section RNA expression could be predicted from the corresponding RNA expression measured in individual colon, together with experimental metadata (histology, groups, DSS treatment and sex). Overall, the models showed poor predictive performance. No model was obtained for *Tnf*, while models for the other seven RNAs generally showed low R^2^ values and poor predictive ability (Q^2^ < 0.5). *Tnfr1* and *Tnfr2* showed better model performance; however, their layer-specific RNA expression did not contribute significantly to the predictions, which were instead mainly driven by experimental metadata.

The second set of multivariate models provided more specific information on RNA expression in the mucosa. We see strong differences in RNA expression between layers that are also RNA-specific. For instance, in the case of *Tnfr1*, the expression levels are the highest in muscularis propria and the lowest in mucosa. For *Duox2*, the expression levels are the highest in the mucosa and the lowest in muscularis propria. For *Tnf*, *Tnfr1*, and *miR-223-3p*, the highest expression levels are observed in the submucosa. In the case of *miR-181a-5p*, the highest expression levels were in the muscularis propria and the lowest in the submucosa. In the case of *miR-3473b*, the highest expression levels were in the mucosa and the lowest in the submucosa. In the mathematical model, this is represented by higher or lower model coefficients for the corresponding layer, meaning that if the sample comes from a specific layer, the expression is elevated or reduced compared to the baseline.

**Figure 7:**
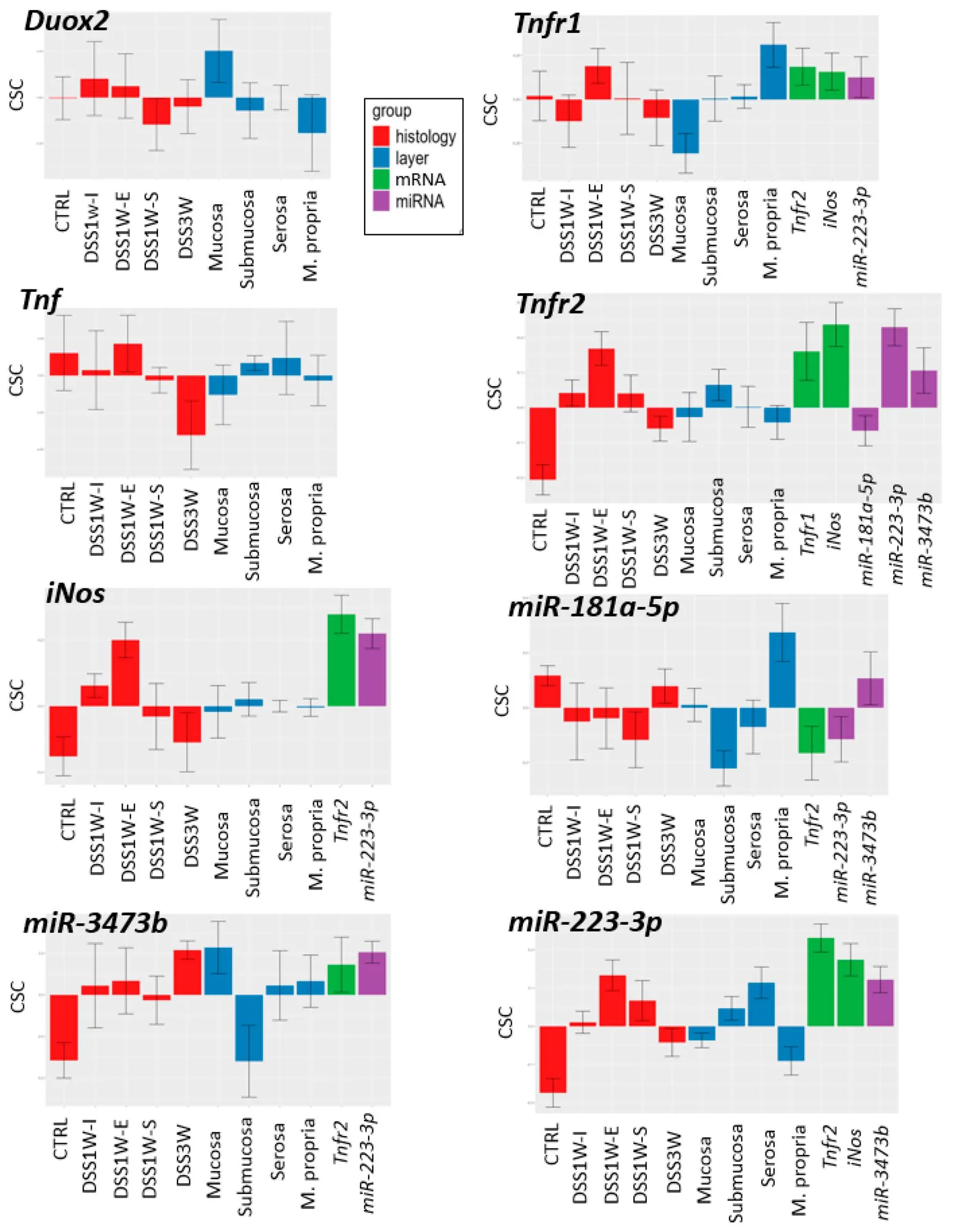
Model-estimated relative contributions of colitis phase, histological phenotype, anatomical layer, and molecular interactions to RNA expression in LMD samples. Each graph shows the relative contributions of the studied factors to the expression level of individual RNAs. Legend: LMD—laser microdissection; CTRL—control group; DSS3W—DSS subacute colitis; DSS1W—DSS acute colitis, from which three different areas were excised (DSS1W-I—inflammation; DSS1W-E—erosion; DSS1W-S—squamous overgrowth); CSC, centred and scaled coefficients.

Using multivariate analyses, we also gained insight into the influence of colitis phase and histological phenotype on RNA expression and into associations among the analyzed molecular factors (Figure 7).

*Tnfr1* expression was positively correlated with *Tnfr2*, *iNos*, and *miR-223-3p*, but not with the other molecular factors. Results show a greater influence of individual layers on *Tnfr1* expression than the histological picture or the stage of inflammation. According to the histological picture, the highest correlation was with erosion (DSS1W). *Tnfr2* expression was positively correlated with the expression of *Tnfr1*, *iNos*, *miR-223-3p* and *miR-3473b* and negatively with *miR-181a-5p*, but not with *Duox2* or *Tnfr2*. Results show a greater influence of the phase and histological phenotype of inflammation on *Tnfr2* expression than of individual colon layers. *Tnfr2* was highly positively correlated with erosion (DSS1W) and negatively correlated with the healthy colon (CTRL). *Tnf* did not correlate with the expression of any of the analyzed molecular factors in the study. For *Tnf*, the relative contributions of the inflammatory phase and histological phenotype were greater than those of the individual layers. The highest correlation of *Tnf* was in erosion (DSS1W), and the lowest in subacute inflammation (DSS3W).

*Duox2* expression did not correlate with any of the analyzed molecular factors in the study, nor with the phase of inflammation. *Duox2* showed the strongest positive correlation with the mucosa (anatomical layer) and a weak negative correlation with squamous overgrowth (histological phenotype). *iNos* expression was positively correlated with *Tnfr2* and *miR-223-3p* expression and negatively with the control group, with the highest positive contribution in erosion and a weak negative contribution in subacute inflammation (DSS3W), but no contribution regarding layers.

*miR-181a-5p* expression was positively correlated with *miR-3473b* and negatively with *Tnfr2* and *miR-223-3p*, with the highest contribution of muscularis propria and the lowest of submucosa (layer), and with the highest positive correlation in the control group and the lowest in squamous overgrowth (histological phenotype).

*miR-223-3p* expression was positively correlated with *Tnfr2*, *iNos*, and *miR-3473b* and significantly influenced by layer (the highest in serosa and submucosa, the lowest in muscularis propria), phase of inflammation (the lowest in control, followed by DSS3W) and histological phenotype of the colitis (the highest in erosion). *miR-3473b* expression was positively correlated with *Tnfr2* and *miR-223-3p*, influenced by the layer (highest in mucosa and lowest in submucosa) and the phase of inflammation (highest in DSS3W inflammation), and negatively correlated with the control group.

Together, these results show that each analyzed RNA has a distinct expression signature in the healthy state that changes in association with inflammatory status, anatomical layer, histological phenotype, colitis phase, and other molecular factors, with particularly pronounced context dependence observed for the miRNAs.

## 4. Discussion

The present study demonstrates that RNA expression measured in whole FFPE colon sections may not fully reflect molecular responses occurring within individual intestinal-wall layers or specific histological microenvironments in DSS-induced colitis. Although several RNAs showed similar overall trends between whole-colon section and spatially defined measurements, substantial differences were observed in expression magnitude, statistical significance, and inter-RNA relationships. Whole-colon section measurements represent composite signals derived from multiple anatomical layers and cell populations and may therefore dilute or obscure spatially restricted molecular responses.

The importance of spatial context is increasingly recognized in human IBD. Single-cell and spatial approaches have demonstrated that inflammatory responses are organized within discrete epithelial, immune, and stromal niches [5,6,7]. Our findings extend this principle to DSS colitis and demonstrate that histology-guided targeted analysis can identify biologically relevant spatial differences even without high-plex spatial technologies. Using LMD, we found that RNA expression varied with both anatomical layer and histological phenotype. This was particularly evident within the mucosa, where inflamed, erosive, and squamous overgrowth regions exhibited distinct molecular profiles, with several inflammatory mediators showing their highest expression in erosive areas. Thus, histological phenotypes appear to represent molecularly distinct microenvironments rather than simply different degrees of inflammation.

These findings provide additional spatial context for gene-expression changes previously reported in DSS colitis. Conventional tissue-based analyses have identified alterations in inflammatory and epithelial barrier-associated genes, including mucins and tight-junction components, but such measurements represent composite signals from the sampled tissue [45,46]. This is particularly relevant because DSS injury itself is spatially heterogeneous: Zolotova et al. recently described a mosaic histological pattern comprising ulcerated (eroded), inflamed but epithelial-intact, and apparently normal regions within the same colon [45]. Recent single-cell and spatial transcriptomic studies further demonstrate that experimental colitis comprises spatially restricted epithelial, immune, and stromal programmes, with distinct molecular states associated with severely damaged or ulcerated (eroded) regions [47,48,49,50]. Consistent with these observations, our histology-guided analysis showed predominant mucosal expression of *Duox2* and particularly pronounced *iNos* expression in erosive mucosa, while *Tnf*, *Tnfr1*, *Tnfr2*, and the investigated miRNAs also showed context-dependent differences among anatomical layers or histological microenvironments. Thus, our findings support and extend previous gene-expression studies by showing that whole-colon measurements may capture overall disease-associated changes while averaging substantial layer- and lesion-specific differences.

At the level of individual targets, *Duox2* showed predominant mucosal expression, consistent with its epithelial localization and role in mucosal defence [26,51], whereas *iNos* was highest in erosive mucosa and correlated with *Tnfr2* and *miR-223-3p*. This pattern is consistent with recent spatial transcriptomic evidence linking *Duox2* and *iNos* to epithelial inflammatory and wound-response programmes in severely damaged DSS-treated colon [49]. The compartment-dependent expression of *Tnfr1* and *Tnfr2* further indicates that TNF signalling, whose receptors have distinct inflammatory, regulatory, and reparative functions [32,33,34], cannot be interpreted independently of tissue context.

Spatial context may be particularly important for interpreting miRNA measurements because miRNA expression can vary among cell types and affected versus unaffected intestinal regions [52]. Moreover, mature miRNAs are not routinely captured by most current single-cell and spatial transcriptomic platforms, making their spatial organization in colitis considerably less characterized than that of mRNAs [53]. *miR-223-3p* was associated with strongly inflamed, immune-cell-rich compartments and correlated with *Tnfr1*, *Tnfr2*, and *iNos*. miR-223 regulates innate immune responses [35], can attenuate DSS colitis through IL-6/STAT3 signalling [38], and its increased expression in IBD has been proposed to represent, at least in part, a compensatory protective response [54]. Our findings therefore suggest that increased *miR-223-3p* may both reflect local inflammatory activity and participate in feedback regulation rather than represent a purely pro-inflammatory response. The context dependence of miRNA function was also apparent for *miR-181a-5p* and *miR-3473b*. The miR-181 family regulates intestinal epithelial inflammation, while *miR-181a-5p* has been implicated in epithelial proliferation and differentiation and in post-transcriptional regulation of TNF [39,55,56]. Such cell-dependent effects may explain the absence of a simple relationship between *miR-181a-5p* and *Tnf* in our dataset. *miR-3473b* correlated with *Tnfr2* and *miR-223-3p* and increased in mucosal and chronic inflammatory contexts. Its role in intestinal inflammation remains poorly characterized; reported involvement in inflammatory and autophagy-related pathways in other tissues [37,57] provides a possible mechanistic context but does not establish an equivalent mechanism in the colon. Our findings therefore identify *miR-3473b* as a candidate component of localized inflammatory or stress responses that warrants further investigation.

An important methodological strength and novel aspect of the present study was the integration of histology-guided spatial sampling with multivariate modelling. The carefully controlled experimental design and the multivariate model enabled us to assess the contributions of histological phenotype, anatomical layer, and colitis phase to variation in RNA expression, while also revealing covariation and interrelationships among the analyzed molecular factors. This integrated analysis provides a level of biological interpretation that is difficult to obtain from conventional univariate or pairwise comparisons alone.

Such spatial and temporal heterogeneity is consistent with the dynamic nature of inflammatory signalling. NF-κB responses encode the position, amplitude, and duration of inflammatory inputs, distinguish between different TNF concentrations, and respond dynamically to changes in cytokine exposure [27,28,58]. This provides a mechanistic framework for understanding why neighbouring tissue compartments exposed to different local inflammatory environments may develop distinct molecular signatures and why a single colon-wide measurement cannot fully represent the molecular state of an inflamed colon.

A further strength of our approach is its applicability to archived FFPE material. FFPE repositories are widely exploited in human pathology and increasingly used for spatial molecular analyses [5,7], whereas archival tissues from animal experiments remain comparatively underused. Previous work established that archived DSS FFPE samples can provide reliable mRNA and miRNA measurements and demonstrated the value of multivariate analysis for identifying experimental and histological factors associated with gene-expression variability [20]. The present study extends this concept by combining archived FFPE material with histology-guided LMD and multivariate modelling, allowing the same tissue resource to be interrogated at spatial, histological, temporal, and molecular-network levels. Although LMD combined with targeted RT-qPCR does not provide the transcriptome-wide resolution of high-plex spatial technologies, it offers a targeted and accessible approach for retrospective spatial analysis of archived FFPE material. Thus, our study shows that FFPE archives from animal experiments contain considerably more biological information than can be extracted by histological assessment or conventional molecular comparisons alone. Integrating experimental metadata, tissue morphology, spatial sampling, molecular measurements, and multivariate analysis allows archived material to be revisited to address new biological questions while increasing the scientific value of existing animal tissue repositories and may reduce the need for additional animal experiments when appropriate archived material is available.

Several limitations should be considered when interpreting these findings and their translational relevance. First, the targeted panel of mRNAs and miRNAs does not provide transcriptome- or proteome-wide characterization, and the spatial patterns observed here cannot be assumed to extend to other molecular pathways. Second, although predefined sample-selection criteria and validated reference genes were used to minimize technical variability associated with archived FFPE material, residual differences in RNA preservation and detection sensitivity cannot be excluded. In addition, because matched fresh-frozen tissue was not available, direct comparison of expression measurements between FFPE and fresh material was not possible. Although previous studies have demonstrated that miRNAs are comparatively robust in FFPE material and that miRNA expression profiles can show good agreement between FFPE and matched fresh-frozen samples [59,60], the present molecular findings should be interpreted specifically within the context of archived FFPE tissue. Third, although the study included both an acute/early post-DSS phase (DSS1W) and a later subacute phase (DSS3W), the DSS model does not reproduce the chronicity and biological complexity of human IBD. In human disease, microbiota, genetic background, environmental exposures, treatment history, disease duration and activity, and other known and potentially unknown factors may further modify spatial molecular patterns and the relationships among molecular factors. Finally, the relatively small number of animals and the use of archived material originating from several independent DSS experiments may limit the robustness and generalizability of the multivariate models, despite the use of predefined selection criteria and inclusion of relevant experimental variables in the analysis. These limitations emphasize the need for future studies using larger datasets, chronic experimental models and human IBD tissue, together with broader spatial transcriptomic or proteomic approaches, to determine the extent to which the spatial patterns and molecular relationships identified here are conserved across experimental and clinical settings.

Importantly, the translational potential of the present approach extends beyond comparison of individual RNA expression patterns. Multivariate analysis enabled the simultaneous evaluation of associations among the investigated molecular factors and their relationships with histological phenotype, anatomical layer, and disease phase. The same analytical concept could be applied to spatially characterized human IBD tissue to determine whether molecular relationships identified under controlled experimental conditions are preserved, modified, or replaced by different associations in human disease. Integration with broader spatial transcriptomic or proteomic profiling could further reveal additional molecular factors and previously unrecognized mechanisms contributing to the greater biological complexity of human IBD.

Taken together, our findings support a model in which DSS-induced colitis comprises spatially and temporally organized inflammatory microenvironments and demonstrates the importance of interpreting molecular measurements within their histological context. Histology-guided LMD provides a complementary, targeted approach to contemporary single-cell and high-plex spatial technologies by preserving the direct relationship between morphology and molecular measurements. Unlike high-plex spatial transcriptomics, LMD-qPCR combined with multivariate analysis (LMD-qPCR-MVA) is designed for targeted rather than transcriptome-wide molecular characterization [61,62]. Spatial transcriptomics provides substantially broader molecular coverage and unbiased discovery potential, enabling the identification of complex cellular states and spatially organized molecular processes [61,63]. In contrast, LMD-qPCR-MVA provides a focused, hypothesis-driven framework for quantifying predefined mRNA and miRNA targets within histologically defined regions of FFPE tissue and integrating their expression with histological, anatomical, and temporal variables through multivariate modelling [62,64]. Thus, the two approaches address distinct but complementary experimental objectives as follows: spatial transcriptomics is particularly suited to broad molecular discovery, whereas LMD-qPCR-MVA enables targeted investigation of candidate molecular signatures within histologically defined tissue regions and can be used to further examine spatial patterns identified by broader profiling approaches.

## 5. Conclusions

In conclusion, our study demonstrates that where and when RNA is measured are critical determinants of its biological interpretation in DSS colitis. Whole FFPE colon sections provide useful overall molecular information. By integrating archived FFPE material, histology-guided LMD, and multivariate modelling, we were able to evaluate spatial, histological, and temporal sources of molecular variation together with interrelationships among the analyzed molecular factors. This integrated approach increases the biological information obtainable from existing tissue repositories and provides a framework for more spatially informed, mechanistically interpretable, and resource-efficient studies of experimental intestinal inflammation.

## Figures and Tables

**Table 1:** Selected mRNA genes and their qPCR amplification characteristics in FFPE colon sections from control and DSS-treated C57BL/6JOlaHsd mice.

						Control Group		DSS Group	
MGI Gene Symbol	Abbreviation Used in Text	Gene Name	Assay ID	Length [bp]	Catalogue No.	Eff. [%]	R^2^	Eff. [%]	R^2^
*Eef2*	*Eef2*	Eucaryotic translation elongation factor 2	Mm01171434_g1	74	4331182	95	0.998	102	0.997
*Rplp0*	*Rplp0*	Ribosomal protein large P0	Mm01974474_gH	89	4331182	89	0.998	91	0.997
*Tnfrsf1a*	*Tnfr1*	Tumour necrosis factor receptor superfamily, member 1a	Mm00441875_m1	69	4331182	98	0.992	99	0.990
*Tnfrsf1b*	*Tnfr2*	Tumour necrosis factor receptor superfamily, member 1b		64	4331182	98	0.994	92	0.994
*Duox2*	*Duox2*	Dual oxidase 2	Mm01326247_m1	65	4331182	96	0.992	99	0.997
*Nos2*	*iNos*	Nitric oxide synthase 2, inducible	Mm00440502_m1	66	4331182	97	0.987	102	0.988
*Tnf*	*Tnf*	Tumour necrosis factor	Mm00443258_m1	81	4331182	75	0.928	99	0.975

**Table 2:** Selected miRNAs and their qPCR amplification characteristics in FFPE colon sections from control and DSS-treated C57BL/6JOlaHsd mice.

				Control Group		DSS Group	
MGI miRNA Gene Symbol(s)	Mature miRNA Analyzed	Assay-Associated miRBase Accession Number	Gene Globe ID/Cat. No.	Eff. [%]	R^2^	Eff. [%]	R^2^
mmu-miR-191-5p	miR-191-5p	MIMAT0000440	YP00204306/339306	109	0.995	107	0.995
mmu-miR-103a-3p	miR-103a-3p	MIMAT0000101	YP00204063/339306	106	0.995	103	0.996
mmu-miR-16-5p	miR-16-5p	MIMAT0000069	YP00205702/339306	100	0.997	96	0.998
mmu-miR-181a-5p	miR-181a-5p	MIMAT0000256	YP00206081/339306	102	0.990	105	0.997
mmu-miR-223-3p	miR-223-3p	MIMAT0000280	YP00205986/339306	101	0.994	93	0.998
mmu-miR-3473b	miR-3473b	MIMAT0020367	YP02111833/ 339306	96	0.999	92	0.998

## Data Availability

Datasets analyzed or generated during the study are available in Supplementary Materials.

## Supplementary Materials

The following supporting information can be downloaded at https://www.mdpi.com/article/10.3390/cells15181677/s1, Figure S1: Representative histological image of FFPE samples from the control group (CTRL, C57BL/6JOlaHsd mice) used for histology-guided laser microdissection and subsequent RNA expression analysis. The upper panel (a) shows an overview of the distal colon and rectum (magnification 4×). Higher-magnification (40×) images show the anus (keratinised stratified squamous epithelium; blue arrow) and rectum–colon junction (brown line) with the transition from squamous to columnar epithelium (b) and histologically normal colon (c). Kreyberg–Jareg staining; Figure S2: Representative histological image of FFPE samples from the acute group (DSS 1W; C57BL/6JOlaHsd mice) used for histology-guided laser microdissection and subsequent RNA expression analysis. The upper panel (a) shows an overview of the distal third of the colon and anus (magnification 4×). Higher-magnification (40×) images: The middle panel (b) shows squamous overgrowth (blue arrow), with stratified squamous epithelium extending from the rectum (brown line) into the adjacent colonic mucosa. Lower panels show erosion (c), characterized by loss of the surface epithelium and crypt architecture with marked inflammatory cell infiltration, and inflammation (d), characterized by preserved epithelial and crypt architecture with mucin depletion and inflammatory cell infiltration. Inflammatory infiltration involves the mucosa and submucosa and is focally transmural. Kreyberg–Jareg staining; Figure S3: Representative histological images of an FFPE colon sample from the subacute group (DSS 3W; C57BL/6JOlaHsd mice), used for histologically-guided laser microdissection and subsequent RNA expression analysis. The upper panel (a; magnification 4×) shows an overview of the distal third of the colon and rectum, with inflammatory cell infiltration of variable severity, predominantly involving the mucosa but extending into the submucosa and focally into deeper layers of the colon wall. Higher-magnification (40×) images: The middle panel (b) shows squamous overgrowth (blue arrow), with stratified squamous epithelium extending from the rectum (brown line) into the adjacent colonic mucosa. The lower panels show histologically heterogeneous areas of inflamed mucosa: an area with sparse residual crypts with marked mucin depletion and dense inflammatory cell infiltration in mucosa (white arrow) and submucosa extending into deeper layers (black arrowhead) (c), and an area with comparatively preserved crypt architecture and mucin content but persistent inflammatory cell infiltration beneath the crypt bases (d; white arrow). Kreyberg–Jareg staining; Figure S4: The figure shows the number of samples collected by laser microdissection (column Colon layer; n) and the number of samples in which RNA expression was above the limit of detection (numbers for each RNA). (a) Total numbers of LMD-collected samples for each colon layer (column Colon layer; n) and samples with detectable RNA expression for each RNA, irrespective of experimental group. (b) Numbers of LMD-collected samples in each layer per group (column Colon layer; n) and samples with detectable RNA expression according to experimental group (CTRL, DSS1W, and DSS3W). (c) Numbers of LMD-collected samples in the DSS1W group for each colon layer (column Colon layer; n) and samples with detectable RNA expression according to histological phenotype: squamous overgrowth (S), inflammation (I), and erosion (E). Legend: n, number of samples collected by LMD: total (a) within a specific group (b) or histological phenotype (c); CTRL, control group; DSS3W, DSS subacute phase; DSS1W, DSS acute phase, (E, erosion; S, squamous overgrowth; I, inflammation); Figure S5: Expression of each RNA (mRNA and miRNA) in mucosa (red box) and in whole-colon sections (blue box) from the control (CTRL) and DSS3W groups (n = 8 C57BL/6JOlaHsd mice; 4 males and 4 females). ΔCq values (y-axis) are shown for each mRNA and miRNA. Legend: CTRL, control group; DSS3W, DSS subacute colitis. Statistical method: Mann–Whitney U test. Statistically significant differences: * *p* < 0.05, ** *p* < 0.01, or *** *p* < 0.001; Figure S6: Expression of each RNA (mRNA and miRNA) in mucosa (red box) and in whole-colon sections (blue box) from the control (CTRL) and DSS 1W groups (n = 8 C57BL/6JOlaHsd mice; 4 males and 4 females). The number of animals does not correspond to the number of samples obtained by laser microdissection. ΔCq values (y-axis) are shown for each mRNA and miRNA. Legend: CTRL, control group; DSS1W, DSS acute colitis, from which three different areas were excised (erosion, squamous overgrowth, inflammation). Statistical method: Mann–Whitney U test. Statistically significant differences: * *p* < 0.05, ** *p* < 0.01, or *** *p* < 0.001; Figure S7: Expression of each RNA (mRNA and miRNA) in inflamed mucosa of the acute (DSS1W) and subacute (DSS3W) groups (n = 8 C57BL/6JOlaHsd mice; 4 males and 4 females). ΔCq values (y-axis) are shown for each mRNA and miRNA. Legend: DSS3W, DSS subacute colitis; and DSS1W, DSS acute colitis. Statistical method: Mann–Whitney U test. Statistically significant differences: * *p* < 0.05, ** *p* < 0.01, or *** *p* < 0.001; Figure S8: The influence of histological phenotypes of colitis on RNA expression in the DSS1W group in mucosa and in whole-colon sections (n = 8 C57BL/6JOlaHsd mice; 4 males and 4 females). ΔCq values for RNA are shown for each histological phenotype (inflammation, erosion, squamous overgrowth). Right-hand side plot shows distributions of α for individual comparisons, which were used to estimate the significance level. The black horizontal line represents α = 0.05 and the red horizontal line represents α = 0.018. Legend: DSS1W (whole-colon), DSS acute colitis, from which three different areas were excised (E, erosion; S, squamous overgrowth; I, inflammation). Statistically significant differences are indicated by * α < 0.018; Table S1: Cross-species sequence conservation and assay specifications for miR-16-5p; Table S2: Cross-species sequence conservation and assay specifications for miR-103a-3p; Table S3: Cross-species sequence conservation and assay specifications for miR-191-5p. Note: The QIAGEN assay is designated according to the human miRNA and therefore reports the human miRBase accession number. The corresponding human and mouse mature miRNAs have distinct miRBase accession numbers but identical mature sequences. According to the manufacturer, the assay targets the corresponding conserved miRNA across multiple species, including *Mus musculus*. All raw data are available from the authors in an Excel file upon reasonable request.

## Abbreviations

The following abbreviations are used in this manuscript:

CD	Crohn’s disease
Cq	quantification cycle
CSC	centred and scaled coefficients
CTRL	control group
DSS	dextran sulfate sodium
DSS1W	acute DSS colitis group
DSS3W	subacute DSS colitis group
FFPE	formalin-fixed paraffin-embedded
IBD	inflammatory bowel disease
LMD	laser microdissection
miRNA	microRNA
mRNA	messenger RNA
PCA	principal component analysis
PLS	partial least squares
RT-qPCR	reverse transcription quantitative polymerase chain reaction
TNF	tumour necrosis factor
TNFR1	tumour necrosis factor receptor 1
TNFR2	tumour necrosis factor receptor 2
UC	ulcerative colitis

## References

[1] Caron B., Honap S., Peyrin-Biroulet L. (2024). Epidemiology of Inflammatory Bowel Disease across the Ages in the Era of Advanced Therapies. J. Crohn’s Colitis.

[2] Guan Q. (2019). A Comprehensive Review and Update on the Pathogenesis of Inflammatory Bowel Disease. J. Immunol. Res..

[3] Cai Z., Wang S., Li J. (2021). Treatment of Inflammatory Bowel Disease: A Comprehensive Review. Front. Med..

[4] Nakase H., Uchino M., Shinzaki S., Matsuura M., Matsuoka K., Kobayashi T., Saruta M., Hirai F., Hata K., Hiraoka S. (2021). Evidence-Based Clinical Practice Guidelines for Inflammatory Bowel Disease 2020. J. Gastroenterol..

[5] Li Y., Wei C., Yang W., Wang H., Robinson A., Peshek I., Yu T., Park J., Ding J.Y., Hanauer S.B. (2026). Spatial Transcriptomics Atlas of Inflammatory Bowel Disease to Guide Implementation in Research Consortiums and Clinical Trials. Nat. Commun..

[6] Kong L., Subramanian S., Segerstolpe Å., Tran V., Shih A.R., Carter G.T., Kunitake H., Twardus S.W., Li J., Gandhi S. (2025). Single-Cell and Spatial Transcriptomics of Stricturing Crohn’s Disease Highlights a Fibrosis-Associated Network. Nat. Genet..

[7] Glyn T., Williams S., Whitehead M., Eglinton T., West N., Purcell R.V. (2024). Digital Spatial Profiling Identifies Molecular Changes Involved in Development of Colitis-Associated Colorectal Cancer. Front. Oncol..

[8] Eichele D.D., Kharbanda K.K. (2017). Dextran Sodium Sulfate Colitis Murine Model: An Indispensable Tool for Advancing Our Understanding of Inflammatory Bowel Diseases Pathogenesis. WJG.

[9] Perše M. (2026). DSS Colitis Model: Traps, Tricks, and Reporting Recommendations. Biomedicines.

[10] Yang C., Merlin D. (2024). Unveiling Colitis: A Journey through the Dextran Sodium Sulfate-Induced Model. Inflamm. Bowel Dis..

[11] Melgar S., Karlsson A., Michaëlsson E. (2005). Acute Colitis Induced by Dextran Sulfate Sodium Progresses to Chronicity in C57BL/6 but Not in BALB/c Mice: Correlation between Symptoms and Inflammation. Am. J. Physiol.-Gastrointest. Liver Physiol..

[12] Perše M., Cerar A. (2012). Dextran Sodium Sulphate Colitis Mouse Model: Traps and Tricks. J. Biomed. Biotechnol..

[13] Bédard A., Westerling-Bui T., Zuraw A. (2021). Proof of Concept for a Deep Learning Algorithm for Identification and Quantification of Key Microscopic Features in the Murine Model of DSS-Induced Colitis. Toxicol. Pathol..

[14] Kobayashi S., Shieh J., Ruiz De Sabando A., Kim J., Liu Y., Zee S.Y., Prasanna P., Bialkowska A.B., Saltz J.H., Yang V.W. (2022). Deep Learning-Based Approach to the Characterization and Quantification of Histopathology in Mouse Models of Colitis. PLoS ONE.

[15] Mähler M., Bristol I.J., Leiter E.H., Workman A.E., Birkenmeier E.H., Elson C.O., Sundberg J.P. (1998). Differential Susceptibility of Inbred Mouse Strains to Dextran Sulfate Sodium-Induced Colitis. Am. J. Physiol.-Gastrointest. Liver Physiol..

[16] Che S., Peng J., Xu X., Wang Y., Huang M., Zhao L., Feng B., Huang P., Li J., Wu M. (2026). Dose-Response Framework for DSS-Induced Colitis in Mice. iScience.

[17] Fransson J., Sorini C., Castillo F., Chi Y., He N., Suarez-Alvarez M., Ulloa M.A., Morales Castro R.A., Okhovat A., Sounart H. (2026). Spatiotemporal Analysis Reveals Distinct Inflammatory Programs Underlying Chronic Colitis. Immunity.

[18] Chassaing B., Aitken J.D., Malleshappa M., Vijay-Kumar M. (2014). Dextran Sulfate Sodium (DSS)-Induced Colitis in Mice. CP Immunol..

[19] Wirtz S., Popp V., Kindermann M., Gerlach K., Weigmann B., Fichtner-Feigl S., Neurath M.F. (2017). Chemically Induced Mouse Models of Acute and Chronic Intestinal Inflammation. Nat. Protoc..

[20] Unkovič A., Boštjančič E., Belič A., Perše M. (2023). Selection and Evaluation of mRNA and miRNA Reference Genes for Expression Studies (qPCR) in Archived Formalin-Fixed and Paraffin-Embedded (FFPE) Colon Samples of DSS-Induced Colitis Mouse Model. Biology.

[21] Brown I.S., Liu C., Miller G.C. (2022). Sampling and Reporting of Inflammatory Bowel Disease. Adv. Anat. Pathol..

[22] Kővári B., Báthori Á., Friedman M.S., Lauwers G.Y. (2022). Histologic Diagnosis of Inflammatory Bowel Diseases. Adv. Anat. Pathol..

[23] Pai R.K., Lauwers G.Y., Pai R.K. (2022). Measuring Histologic Activity in Inflammatory Bowel Disease: Why and How. Adv. Anat. Pathol..

[24] Bevilacqua C., Ducos B. (2018). Laser Microdissection: A Powerful Tool for Genomics at Cell Level. Mol. Asp. Med..

[25] Iacomino G., Rotondi Aufiero V., Iannaccone N., Melina R., Giardullo N., De Chiara G., Venezia A., Taccone F.S., Iaquinto G., Mazzarella G. (2020). IBD: Role of Intestinal Compartments in the Mucosal Immune Response. Immunobiology.

[26] Sommer F., Bäckhed F. (2015). The Gut Microbiota Engages Different Signaling Pathways to Induce Duox2 Expression in the Ileum and Colon Epithelium. Mucosal Immunol..

[27] Son M., Frank T., Holst-Hansen T., Wang A.G., Junkin M., Kashaf S.S., Trusina A., Tay S. (2022). Spatiotemporal NF-kB Dynamics Encodes the Position, Amplitude, and Duration of Local Immune Inputs. Sci. Adv..

[28] Zhang Q., Gupta S., Schipper D.L., Kowalczyk G.J., Mancini A.E., Faeder J.R., Lee R.E.C. (2017). NF-κB Dynamics Discriminate between TNF Doses in Single Cells. Cell Syst..

[29] Mu K., Yu S., Kitts D.D. (2019). The Role of Nitric Oxide in Regulating Intestinal Redox Status and Intestinal Epithelial Cell Functionality. Int. J. Mol. Sci..

[30] Soufli I., Toumi R., Rafa H., Touil-Boukoffa C. (2016). Overview of Cytokines and Nitric Oxide Involvement in Immuno-Pathogenesis of Inflammatory Bowel Diseases. World J. Gastrointest. Pharmacol. Ther..

[31] Wei X., Xue M., Kang C., Gao L., Zhang M., Ma C., Jia W., Zheng Y., Cao L., Chen P. (2022). Increased NOX1 and DUOX2 Expression in the Colonic Mucosa of Patients with Chronic Functional Constipation. Medicine.

[32] Naserian S., Abdelgawad M.E., Afshar Bakshloo M., Ha G., Arouche N., Cohen J.L., Salomon B.L., Uzan G. (2020). The TNF/TNFR2 Signaling Pathway Is a Key Regulatory Factor in Endothelial Progenitor Cell Immunosuppressive Effect. Cell Commun. Signal.

[33] Steeland S., Libert C., Vandenbroucke R.E. (2018). A New Venue of TNF Targeting. Int. J. Mol. Sci..

[34] Yang S., Wang J., Brand D.D., Zheng S.G. (2018). Role of TNF–TNF Receptor 2 Signal in Regulatory T Cells and Its Therapeutic Implications. Front. Immunol..

[35] Jiao P., Wang X.-P., Luoreng Z.-M., Yang J., Jia L., Ma Y., Wei D.-W. (2021). miR-223: An Effective Regulator of Immune Cell Differentiation and Inflammation. Int. J. Biol. Sci..

[36] Lee J., Park E.J., Yuki Y., Ahmad S., Mizuguchi K., Ishii K.J., Shimaoka M., Kiyono H. (2015). Profiles of microRNA Networks in Intestinal Epithelial Cells in a Mouse Model of Colitis. Sci. Rep..

[37] Wang X., Chen S., Ni J., Cheng J., Jia J., Zhen X. (2018). miRNA-3473b Contributes to Neuroinflammation Following Cerebral Ischemia. Cell Death Dis..

[38] Zhang J., Wang C., Guo Z., Da B., Zhu W., Li Q. (2021). miR-223 Improves Intestinal Inflammation through Inhibiting the IL-6/STAT3 Signaling Pathway in Dextran Sodium Sulfate-induced Experimental Colitis. Immun. Inflam. Dis..

[39] Zhu J., Wang F.-L., Wang H.-B., Dong N., Zhu X.-M., Wu Y., Wang Y.-T., Yao Y.-M. (2017). TNF-α mRNA Is Negatively Regulated by microRNA-181a-5p in Maturation of Dendritic Cells Induced by High Mobility Group Box-1 Protein. Sci. Rep..

[40] Latham G.J., Monticelli S. (2010). Normalization of MicroRNA Quantitative RT-PCR Data in Reduced Scale Experimental Designs. MicroRNAs and the Immune System.

[41] Benjamini Y., Hochberg Y. (1995). Controlling the False Discovery Rate: A Practical and Powerful Approach to Multiple Testing. J. R. Stat. Soc. Ser. B Stat. Methodol..

[42] Kramer R. (1998). Chemometric Techniques for Quantitative Analysis.

[43] Wold S., Sjöström M., Eriksson L. (2001). PLS-Regression: A Basic Tool of Chemometrics. Chemom. Intell. Lab. Syst..

[44] Liu C.Y., Girish N., Gomez M.L., Dubé P.E., Washington M.K., Simons B.D., Polk D.B. (2022). Transitional Anal Cells Mediate Colonic Re-Epithelialization in Colitis. Gastroenterology.

[45] Zolotova N.A., Kirillova M.V., Tsvetkov I.S., Dzhalilova D.S., Ozeretskaya L.V., Dobrynina M.T., Makarova O.V. (2025). The Morphofunctional Profile of a Mouse Model of Acute Moderate Dextran Sodium Sulfate-Induced Colitis. Mosc. Univ. Biol.Sci. Bull..

[46] Zolotova N.A., Polikarpova A.V., Khochanskii D.N., Makarova O.V., Mikhailova L.P. (2018). Expression of Mucins and Claudins in the Colon during Acute and Chronic Experimental Colitis. Bull. Exp. Biol. Med..

[47] Parigi S.M., Larsson L., Das S., Ramirez Flores R.O., Frede A., Tripathi K.P., Diaz O.E., Selin K., Morales R.A., Luo X. (2022). The Spatial Transcriptomic Landscape of the Healing Mouse Intestine Following Damage. Nat. Commun..

[48] Cadinu P., Sivanathan K.N., Misra A., Xu R.J., Mangani D., Yang E., Rone J.M., Tooley K., Kye Y.-C., Bod L. (2024). Charting the Cellular Biogeography in Colitis Reveals Fibroblast Trajectories and Coordinated Spatial Remodeling. Cell.

[49] Mayassi T., Li C., Segerstolpe Å., Brown E.M., Weisberg R., Nakata T., Yano H., Herbst P., Artis D., Graham D.B. (2024). Spatially Restricted Immune and Microbiota-Driven Adaptation of the Gut. Nature.

[50] Yalom L.K., Herrnreiter C.J., Bui T.M., Lockhart J., Piccolo E.B., Ren X., Wei C., Serdiukova A., Thorp E.B., Dulai P.S. (2025). Spatially Separated Epithelium-Associated and Lamina Propria Neutrophils Present Distinct Functional Identities in the Inflamed Colon Mucosa. Mucosal Immunol..

[51] Csillag C., Haagen Nielsen O., Vainer B., Olsen J., Dieckgraefe B.K., Hendel J., Vind I., Dupuy C., Cilius Nielsen F., Borup R. (2007). Expression of the Genes Dual Oxidase 2, Lipocalin 2 and Regenerating Islet-Derived 1 Alpha in Crohn’s Disease. Scand. J. Gastroenterol..

[52] Mohammadi A., Kelly O.B., Smith M.I., Kabakchiev B., Silverberg M.S. (2019). Differential miRNA Expression in Ileal and Colonic Tissues Reveals an Altered Immunoregulatory Molecular Profile in Individuals with Crohn’s Disease versus Healthy Subjects. J. Crohn’s Colitis.

[53] Robles-Remacho A., Zou Y., Grillo M., Nilsson M. (2025). Spatially Resolved microRNA Expression in Tissues: Technologies, Challenges, and Opportunities. Trends Genet..

[54] Chen J., Vitetta L. (2023). Is miR-223 Upregulation in Inflammatory Bowel Diseases a Protective Response?. Front. Biosci. (Elite Ed.).

[55] Jimenez M.T., Clark M.L., Wright J.M., Michieletto M.F., Liu S., Erickson I., Dohnalova L., Uhr G.T., Tello-Cajiao J., Joannas L. (2022). The miR-181 Family Regulates Colonic Inflammation through Its Activity in the Intestinal Epithelium. J. Exp. Med..

[56] Li C., Zhou Y., Yin Z., Jiang Y., Liu J., Weiss H.L., Wang Q., Evers B.M. (2025). miR-181a-5p Mediates the Effects of BMP4 on Intestinal Cell Proliferation and Differentiation. Cell Death Dis..

[57] Lv Q., Zhong Z., Hu B., Yan S., Yan Y., Zhang J., Shi T., Jiang L., Li W., Huang W. (2021). MicroRNA-3473b Regulates the Expression of TREM2/ULK1 and Inhibits Autophagy in Inflammatory Pathogenesis of Parkinson Disease. J. Neurochem..

[58] Son M., Wang A.G., Tu H.-L., Metzig M.O., Patel P., Husain K., Lin J., Murugan A., Hoffmann A., Tay S. (2021). NF-κB Responds to Absolute Differences in Cytokine Concentrations. Sci. Signal..

[59] Li J., Smyth P., Flavin R., Cahill S., Denning K., Aherne S., Guenther S.M., O’Leary J.J., Sheils O. (2007). Comparison of miRNA Expression Patterns Using Total RNA Extracted from Matched Samples of Formalin-Fixed Paraffin-Embedded (FFPE) Cells and Snap Frozen Cells. BMC Biotechnol..

[60] Xi Y., Nakajima G., Gavin E., Morris C.G., Kudo K., Hayashi K., Ju J. (2007). Systematic Analysis of microRNA Expression of RNA Extracted from Fresh Frozen and Formalin-Fixed Paraffin-Embedded Samples. RNA.

[61] Rao A., Barkley D., França G.S., Yanai I. (2021). Exploring Tissue Architecture Using Spatial Transcriptomics. Nature.

[62] Bidarimath M., Edwards A.K., Tayade C., Mor G., Alvero A.B. (2015). Laser Capture Microdissection for Gene Expression Analysis. Apoptosis and Cancer.

[63] Gulati G.S., D’Silva J.P., Liu Y., Wang L., Newman A.M. (2025). Profiling Cell Identity and Tissue Architecture with Single-Cell and Spatial Transcriptomics. Nat. Rev. Mol. Cell Biol..

[64] Paulsen S.J., Larsen L.K., Merighi A. (2011). Laser Capture Microdissection and Quantitative-PCR Analysis. Neuropeptides.

