# Supplementary material for: Colon Layer and Histological Phenotype Influence mRNA and miRNA Expression in Colon Samples of the DSS Mouse Model
Source: Cells. 2026 Sep 16;15(18):1677. doi: 10.3390/cells15181677 (PMC13605476; doi:10.3390/cells15181677)
Supplement: Supplementary file 1 [file cells-15-01677-s001.zip › cells-4524063-supplementary.pdf]

Supplementary Tables:

Supplementary information on cross-species applicability of miRNA assays

Table S1. Cross-species sequence conservation and assay specifications for miR-16-5p

Parameter	Human / QIAGEN assay	Mouse
**Mature miRNA**	hsa-miR-16-5p	mmu-miR-16-5p
**miRBase accession**	MIMAT0000069	MIMAT0000527
**Mature sequence (miRBASE)**	UAGCAGCACGUAAAUAUUGGCG	UAGCAGCACGUAAAUAUUGGCG
**Sequence identity**	**Identical (100%)**	**Identical (100%)**
**QIAGEN assay name**	hsa-miR-16-5p	—
**QIAGEN assay ID**	YP00205702	—
**Assay specification by QIAGEN**	Pre-designed assay for qPCR and dPCR; wet-lab verified; targets similar miRNAs across multiple species, including *Mus musculus*	—
**Alternative target specified by QIAGEN**	mmu-miR-16-5p	—
**Target sequence by QIAGEN**	**UAGCAGCACGUAAAUAUUGGCG**	

Note: The QIAGEN assay is designated according to the human miRNA and therefore reports the human miRBase accession number. The corresponding human and mouse mature miRNAs have distinct miRBase accession numbers but identical mature sequences. According to the manufacturer, the assay targets the corresponding conserved miRNA across multiple species, including *Mus musculus.*

Table S2. Cross-species sequence conservation and assay specifications for miR-103a-3p

Parameter	Human / QIAGEN assay	Mouse
**Mature miRNA**	hsa-miR-103a-3p	mmu-miR-103-3p
**miRBase accession**	MIMAT0000101	MIMAT0000546
**Mature sequence (miRBASE)**	AGCAGCAUUGUACAGGGCUAUGA	AGCAGCAUUGUACAGGGCUAUGA
**Sequence identity**	**Identical (100%)**	**Identical (100%)**
**QIAGEN assay name**	hsa-miR-103a-3p	—
**QIAGEN assay ID**	YP00204063	—
**Assay specification by QIAGEN**	Pre-designed assay for qPCR and dPCR; wet-lab verified; targets similar miRNAs across multiple species, including *Mus musculus*	—
**Alternative target specified by QIAGEN**	mmu-miR-103-3p	—

**Target sequence by QIAGEN**	**AGCAGCAUUGUACAGGGCUAUGA**

Note: The QIAGEN assay is designated according to the human miRNA and therefore reports the human miRBase accession number. The corresponding human and mouse mature miRNAs have distinct miRBase accession numbers but identical mature sequences. According to the manufacturer, the assay targets the corresponding conserved miRNA across multiple species, including *Mus musculus.*

Table S3. Cross-species sequence conservation and assay specifications for miR-191-5p

Parameter	Human / QIAGEN assay	Mouse
**Mature miRNA**	hsa-miR-191-5p	mmu-miR-191-5p
**miRBase accession**	MIMAT0000440	MIMAT0000221
**Mature sequence (miRBASE)**	CAACGGAAUCCCAAAAGCAGCUG	CAACGGAAUCCCAAAAGCAGCUG
**Sequence identity**	**Identical (100%)**	**Identical (100%)**
**QIAGEN assay name**	hsa-miR-191-5p	—
**QIAGEN assay ID**	YP00204306	—
**Assay specification by QIAGEN**	Pre-designed assay for qPCR and dPCR; wet-lab verified; targets similar miRNAs across multiple species, including *Mus musculus*	—
**Alternative target specified by QIAGEN**	mmu-miR-191-5p	—
**Target sequence by QIAGEN**	**CAACGGAAUCCCAAAAGCAGCUG**	

Note: The QIAGEN assay is designated according to the human miRNA and therefore reports the human miRBase accession number. The corresponding human and mouse mature miRNAs have distinct miRBase accession numbers but identical mature sequences. According to the manufacturer, the assay targets the corresponding conserved miRNA across multiple species, including *Mus musculus.*

Supplementary Figures:

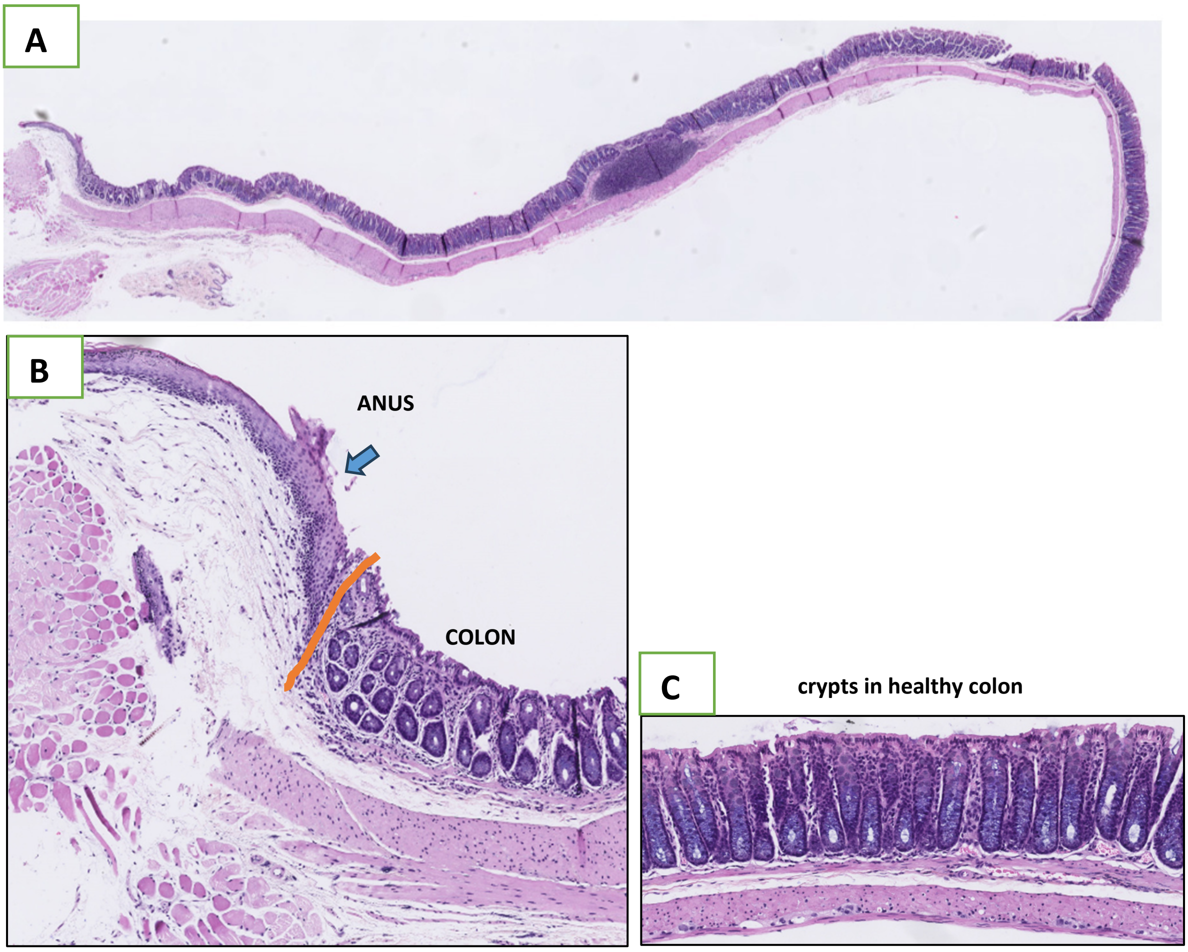

Figure S1. Representative histological image of FFPE samples from the control group (CTRL, C57BL/6JOlaHsd mice) used for histology-guided laser microdissection and subsequent RNA expression analysis. The upper panel (a) shows an overview of the distal colon and rectum (magnification 4x). Higher-magnification (40x) images show the anus (keratinised stratified squamous epithelium; blue arrow) and rectum–colon junction (brown line) with the transition from squamous to columnar epithelium (b) and histologically normal colon (c). **Kreyberg–Jareg staining.**

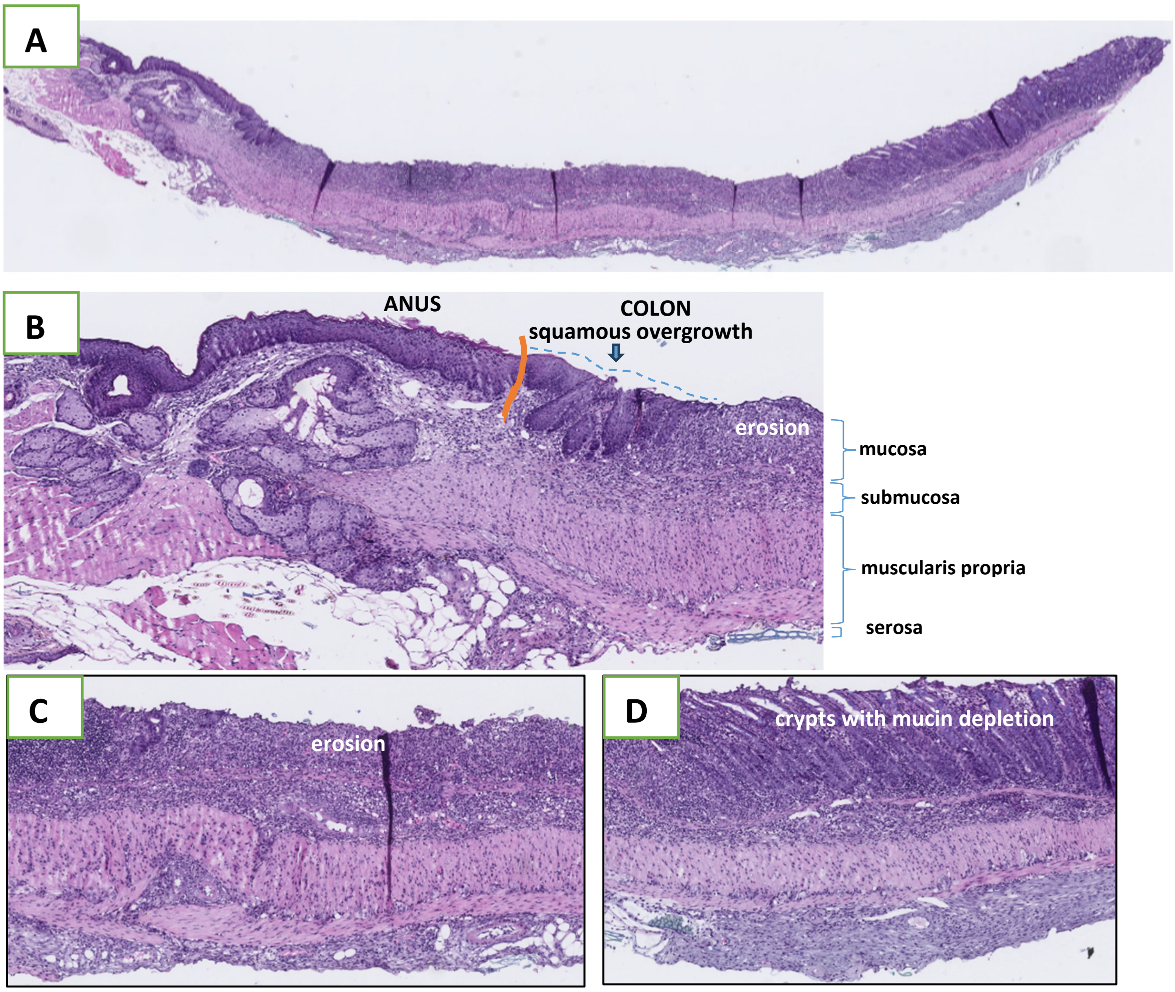

Figure S2. Representative histological image of FFPE samples from the acute group (DSS 1W; C57BL/6JOlaHsd mice) used for histology-guided laser microdissection and subsequent RNA expression analysis. The upper panel (a) shows an overview of the distal third of the colon and anus (magnification 4x). Higher-magnification (40x) images: The middle panel (b) shows **squamous overgrowth (blue arrow)**, with stratified squamous epithelium extending from the rectum (brown line) into the adjacent colonic mucosa. Lower panels show **erosion** (c), characterized by loss of the surface epithelium and crypt architecture with marked inflammatory cell infiltration, and **inflammation** (d), characterized by preserved epithelial and crypt architecture with mucin depletion and inflammatory cell infiltration. Inflammatory infiltration involves the mucosa and submucosa and is focally transmural. **Kreyberg–Jareg staining.**

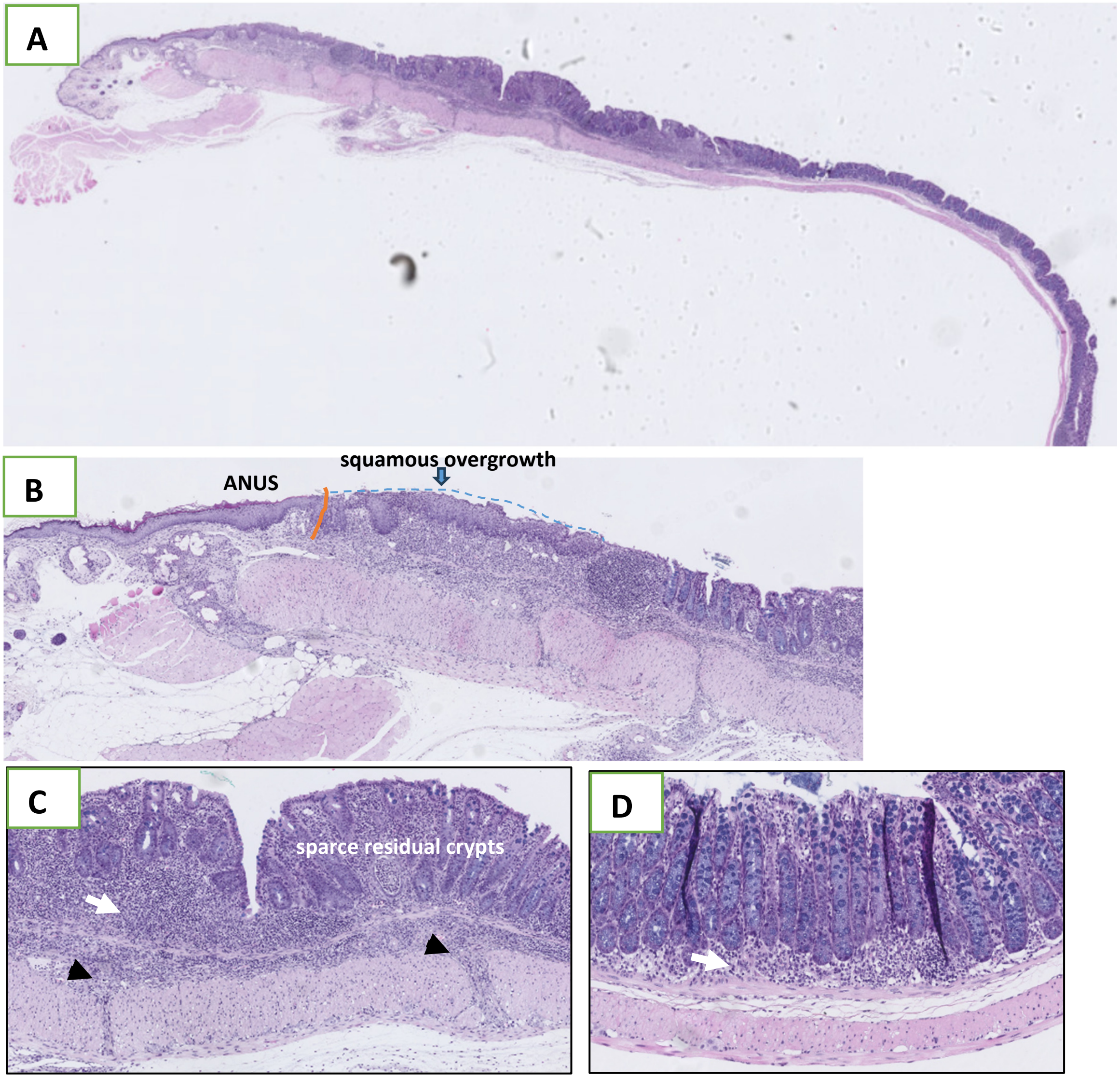

Figure S3. Representative histological images of an FFPE colon sample from the subacute group (DSS 3W; C57BL/6JOlaHsd mice), used for histologically-guided laser microdissection and subsequent RNA expression analysis. The upper panel (a; magnification 4x) shows an overview of the distal third of the colon and rectum, with inflammatory cell infiltration of variable severity, predominantly involving the mucosa but extending into the submucosa and focally into deeper layers of the colon wall. Higher-magnification (40x) images: The middle panel (b) shows **squamous overgrowth (blue arrow)**, with stratified squamous epithelium extending from the rectum (brown line) into the adjacent colonic mucosa. The lower panels show histologically heterogeneous areas of inflamed mucosa: an area with sparse residual crypts with marked mucin depletion and dense inflammatory cell infiltration in mucosa (white arrow) and submucosa extending into deeper layers (black arrowhead) (c), and an area with comparatively preserved crypt architecture and mucin content but persistent inflammatory cell infiltration beneath the crypt bases (d; white arrow). **Kreyberg–Jareg staining.**

a

Colon layer\Mol. factor	*miR-223-3p*	*miR-181a-5p*	*miR-3473b*	*Tnfr1*	*Tnfr2*	*Duox2*	*iNos*	*Tnf*
**Mucose (all, n=40)**	**40**	**40**	**40**	**39**	**40**	**38**	**37**	**27**
**Submucose (all, n=40)**	**40**	**31**	**35**	**7**	**7**	**2**	**2**	**3**
**M. propria (all, n=32)**	**32**	**32**	**32**	**22**	**13**	**5**	**1**	**3**
**Serose (all, n=13)**	**13**	**12**	**13**	**3**	**2**	**0**	**0**	**1**
**Whole-colon sections (all, n=24)**	**24**	**24**	**24**	**24**	**24**	**24**	**24**	**24**

b

	Colon layer\Mol. factor	*miR-223-3p*	*miR-181a-5p*	*miR-3473b*	*Tnfr1*	*Tnfr2*	*Duox2*	*iNos*	*Tnf*
Mucose	Mucose (CTRL, n=8)	8	8	8	8	8	8	7	2
	Mucose (DSS1W, n=24)	24	24	24	23	24	22	22	18
	Mucose (DSS3W, n=8)	8	8	8	8	8	8	8	7
Submucose	Submucose (CTRL, n=8)	8	4	5	0	0	0	0	0
	Submucose (DSS1W, n=24)	24	20	23	7	7	2	2	3
	Submucose (DSS3W, n=8)	8	7	8	0	0	0	0	0
M. propria	M. propria (CTRL, n=8)	8	8	8	5	3	3	0	0
	M. propria (DSS1W, n=16)	16	16	16	9	6	0	1	2
	M. propria (DSS3W, n=8)	8	8	8	8	4	2	0	1
Serose	Serose (DSS1W, n=13)	13	12	13	3	2	0	0	1
Whole-colon sections	Whole-colon (CTRL, n=8)	8	8	8	8	8	8	8	8
	Whole-colon (DSS1W, n=8)	8	8	8	8	8	8	8	8
	Whole-colon (DSS 3W, n=8)	8	8	8	8	8	8	8	8

c

		Colon layer\Mol. factor	*miR-223-3p*	*miR-181a-5p*	*miR-3473b*	*Tnfr1*	*Tnfr2*	*Duox2*	*iNos*	*Tnf*
DSS1W	Mucose	Mucose (DSS1W-S, n=8)	8	8	8	7	8	7	6	4
		Mucose (DSS1W-E, n=8)	8	8	8	8	8	7	8	8
		Mucose (DSS1W-I, n=8)	8	8	8	8	8	8	8	6
	Submucose	Submucose (DSS1W-S, n=8)	8	8	8	3	2	1	0	0
		Submucose (DSS1W-E, n=8)	8	7	8	3	3	0	2	2
		Submucose (DSS1W-I, n=8)	8	5	7	1	2	1	0	1
	M. propria	M. propria (DSS1W-E, n=8)	8	8	8	5	4	0	0	2
		M. propria (DSS1W-I, n=8)	8	8	8	4	2	0	1	0
	Serose	Serose (DSS1W-E, n=8)	8	7	8	2	1	0	0	0
		Serose (DSS1W-I, n=8)	5	5	5	1	1	0	0	1

Figure S4. The figure shows the number of samples collected by laser microdissection (column Colon layer; n) and the number of samples in which RNA expression was above the limit of detection (numbers for each RNA). **(a)** Total numbers of LMD-collected samples for each colon layer (column Colon layer; n) and samples with detectable RNA expression for each RNA, irrespective of experimental group. **(b)** Numbers of LMD-collected samples in each layer per group (column Colon layer; n) and samples with detectable RNA expression according to experimental group (CTRL, DSS1W, and DSS3W). **(c)** Numbers of LMD-collected samples in the DSS1W group for each colon layer (column Colon layer; n) and samples with detectable RNA expression according to histological phenotype: squamous overgrowth (S), inflammation (I), and erosion (E). **Legend**: n, number of samples collected by LMD: total (a) within a specific group (b) or histological phenotype (c); CTRL, control group; DSS3W, DSS subacute phase; DSS1W, DSS acute phase, (E, erosion; S, squamous overgrowth; I, inflammation).

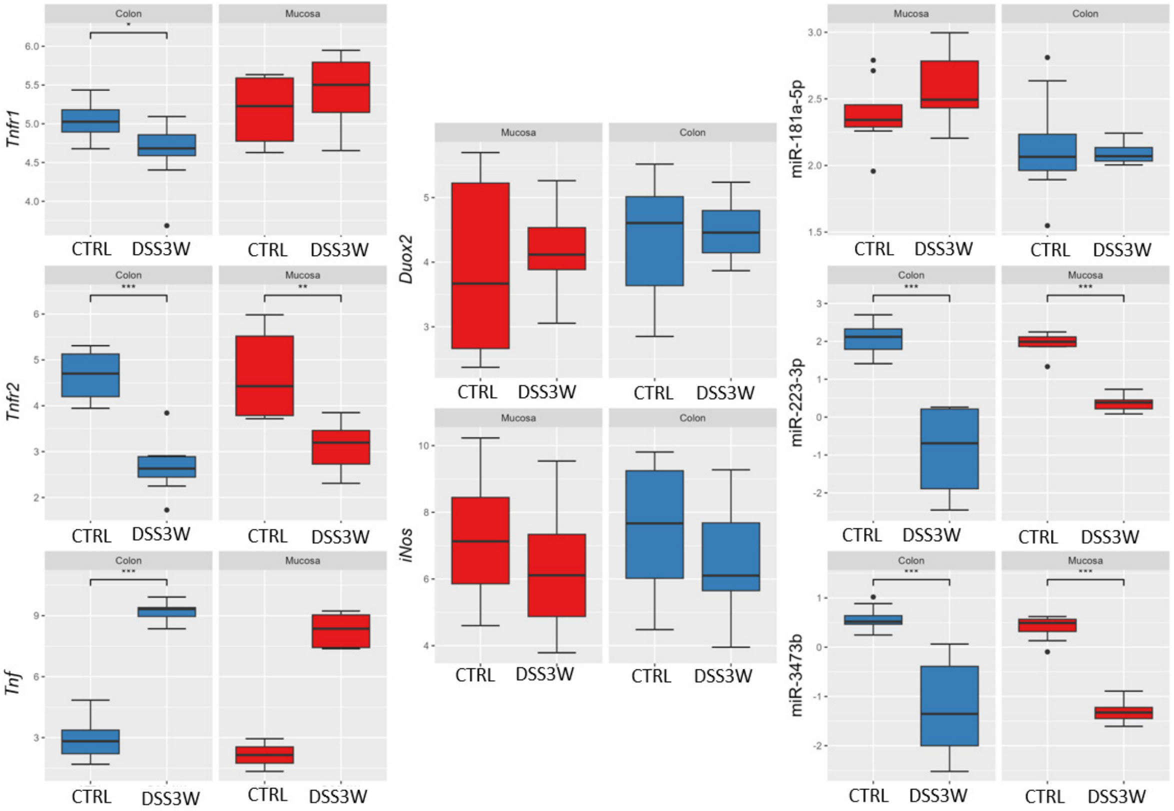

Figure S5. Expression of each RNA (mRNA and miRNA) in mucosa (red box) and in whole-colon sections (blue box) from the control (CTRL) and DSS3W groups (n=8 C57BL/6JOlaHsd mice; 4 males and 4 females). ΔCq values (y-axis) are shown for each mRNA and miRNA. Legend: CTRL, control group; DSS3W, DSS subacute colitis. Statistical method: Mann–Whitney U test. Statistically significant differences: * $p < 0.05$, ** $p < 0.01$, or *** $p < 0.001$.

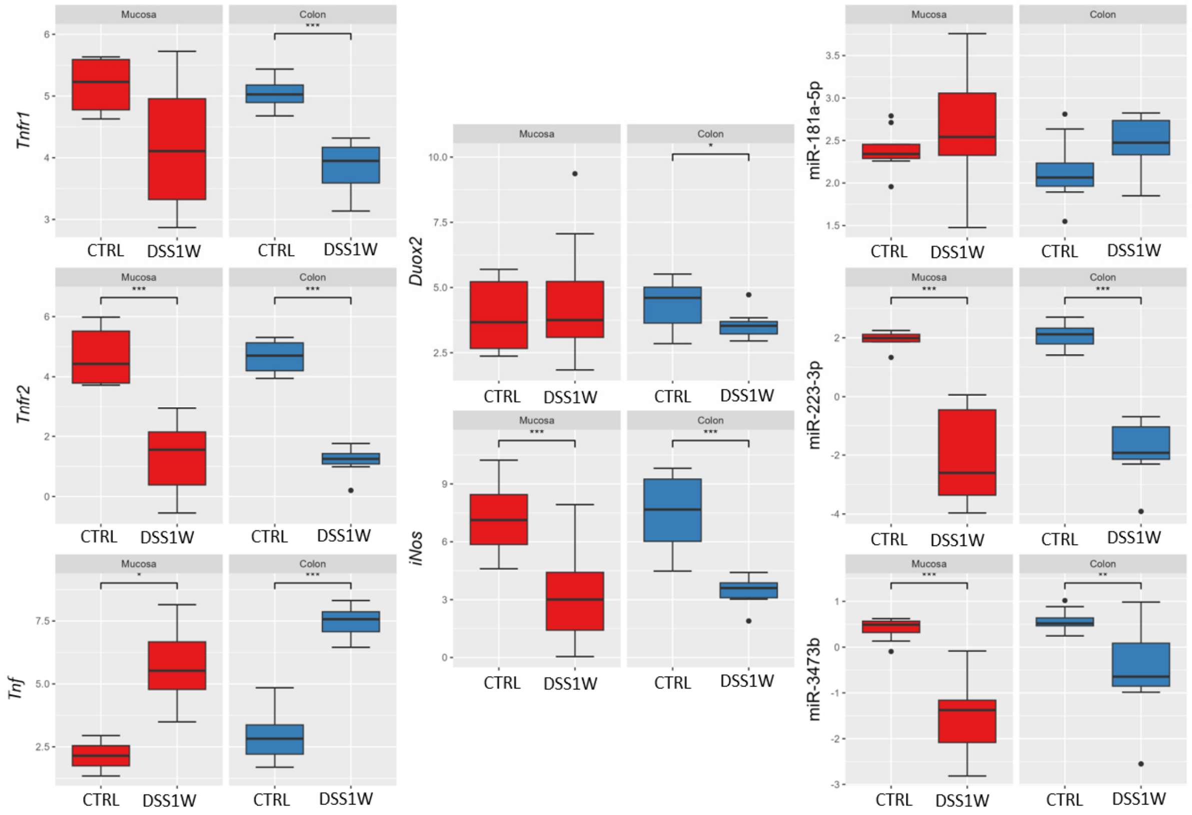

Figure S6. Expression of each RNA (mRNA and miRNA) in mucosa (red box) and in whole-colon sections (blue box) from the control (CTRL) and DSS 1W groups (n=8 C57BL/6JOlaHsd mice; 4 males and 4 females). The number of animals does not correspond to the number of samples obtained by laser microdissection. ΔCq values (y-axis) are shown for each mRNA and miRNA. Legend: CTRL, control group; DSS1W, DSS acute colitis, from which three different areas were excised (erosion, squamous overgrowth, inflammation). Statistical method: Mann–Whitney U test. Statistically significant differences: * $p < 0.05$, ** $p < 0.01$, or *** $p < 0.001$.

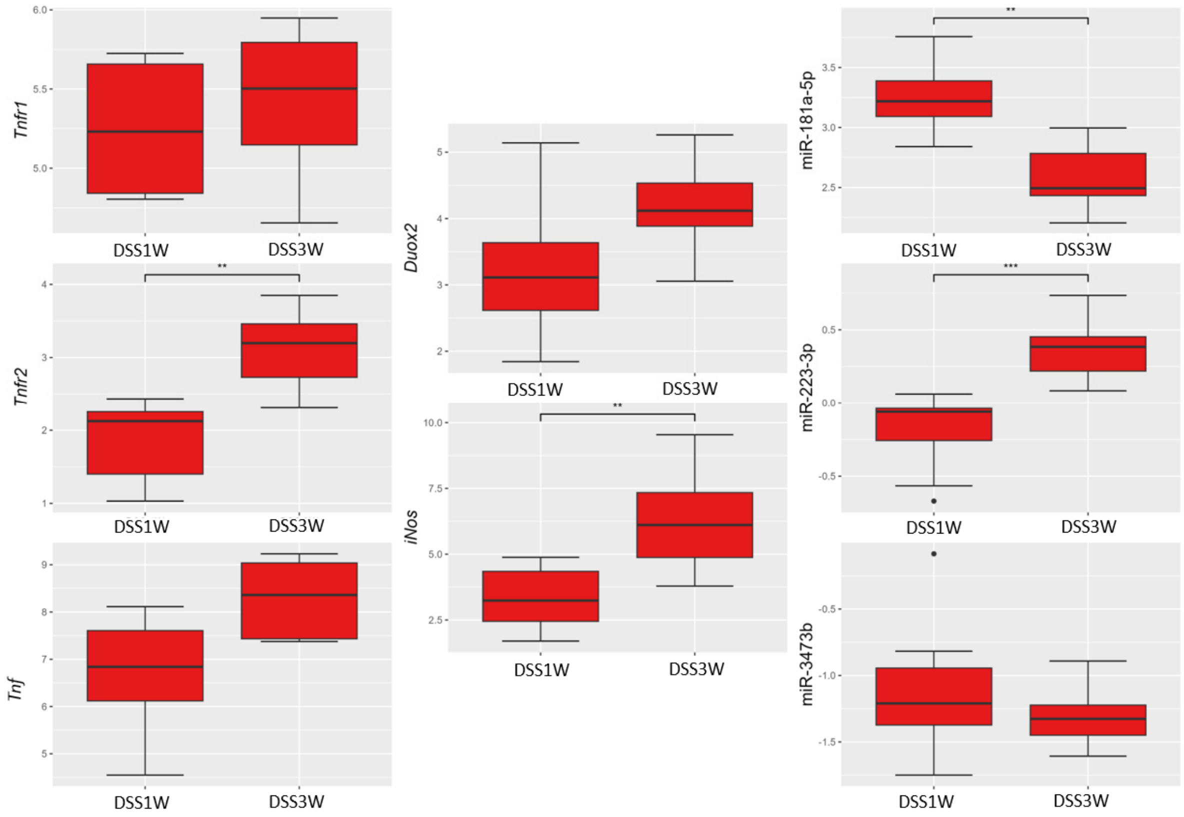

Figure S7. Expression of each RNA (mRNA and miRNA) in inflamed mucosa of the acute (DSS1W) and subacute (DSS3W) groups (n=8 C57BL/6JOlaHsd mice; 4 males and 4 females). ΔCq values (y-axis) are shown for each mRNA and miRNA. Legend: DSS3W, DSS subacute colitis; and DSS1W, DSS acute colitis. Statistical method: Mann–Whitney U test. Statistically significant differences: * $p < 0.05$, ** $p < 0.01$, or *** $p < 0.001$.

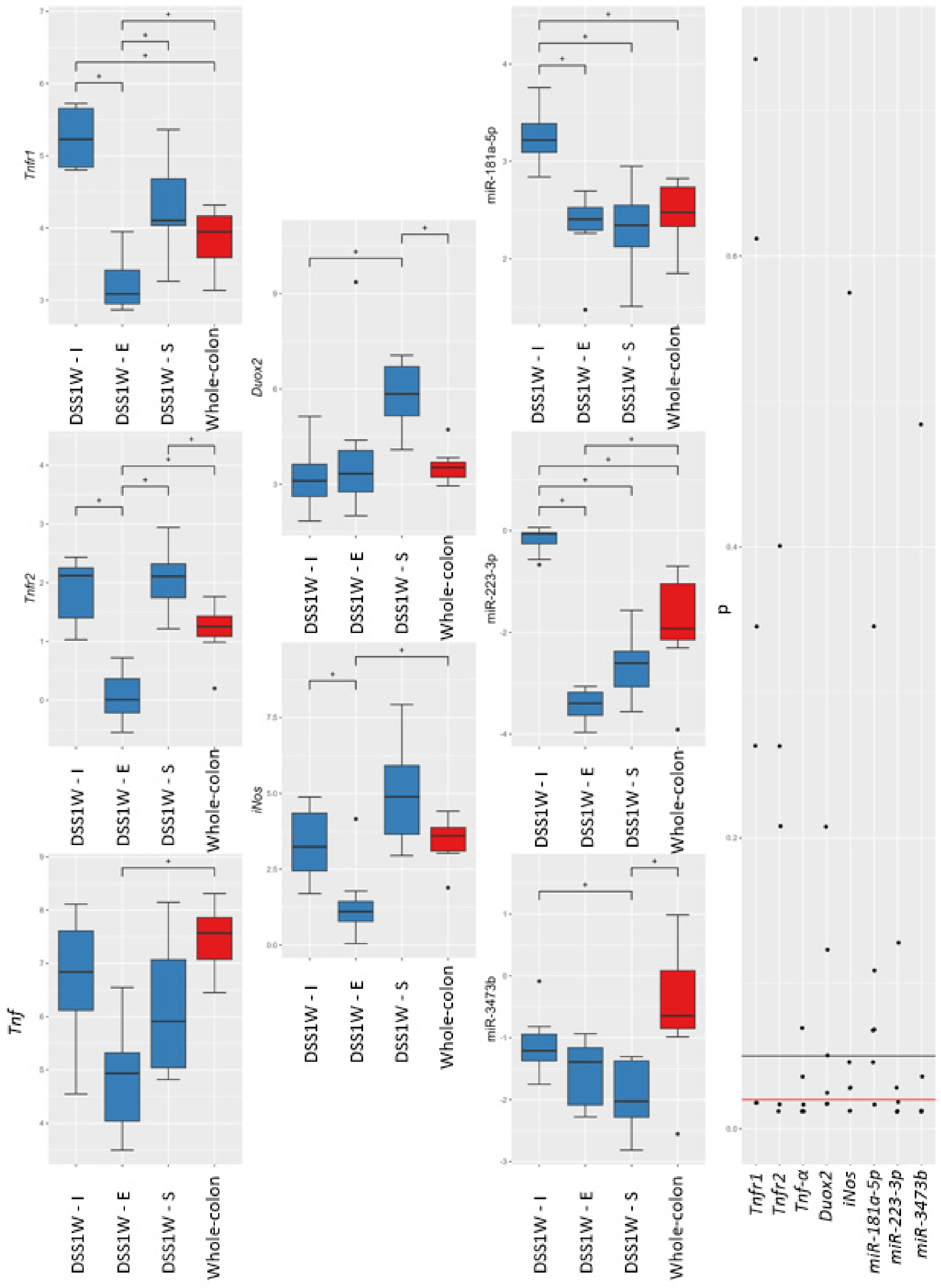

Figure S8. The influence of histological phenotypes of colitis on RNA expression in the DSS1W group in mucosa and in whole-colon sections (n=8 C57BL/6JOlaHsd mice; 4 males and 4 females). ΔCq values for RNA are shown for each histological phenotype (inflammation, erosion, squamous overgrowth). Right-hand side plot shows distributions of α for individual comparisons, which were used to estimate the significance level. The black horizontal line represents α=0.05 and the red horizontal line represents α=0.018. Legend: DSS1W (whole-colon), DSS acute colitis, from which three different areas were excised (E, erosion; S, squamous overgrowth; I, inflammation). Statistically significant differences are indicated by * α<0.018.
